# Standing genetic variation in laboratory populations of insecticide‐susceptible *Phlebotomus papatasi* and *Lutzomyia longipalpis* (Diptera: Psychodidae: Phlebotominae) for the evolution of resistance

**DOI:** 10.1111/eva.13194

**Published:** 2021-02-09

**Authors:** David S. Denlinger, Spencer B. Hudson, Nadya S. Keweshan, Zachariah Gompert, Scott A. Bernhardt

**Affiliations:** ^1^ Department of Biology Utah State University Logan UT USA

**Keywords:** genome‐wide association mapping studies, genotype‐by‐sequencing, insecticide, leishmaniasis, *Lutzomyia longipalpis*, *Phlebotomus papatasi*, resistance

## Abstract

Insecticides can exert strong selection on insect pest species, including those that vector diseases, and have led to rapid evolution of resistance. Despite such rapid evolution, relatively little is known about standing genetic variation for resistance in insecticide‐susceptible populations of many species. To help fill this knowledge gap, we generated genotyping‐by‐sequencing data from insecticide‐susceptible *Phlebotomus papatasi* and *Lutzomyia longipalpis* sand flies that survived or died from a sub‐diagnostic exposure to either permethrin or malathion using a modified version of the Centers for Disease Control and Prevention bottle bioassay. Multi‐locus genome‐wide association mapping methods were used to quantify standing genetic variation for insecticide resistance in these populations and to identify specific alleles associated with insecticide survival. For each insecticide treatment, we estimated the proportion of the variation in survival explained by the genetic data (i.e., “chip” heritability) and the number and contribution of individual loci with measurable effects. For all treatments, survival to an insecticide exposure was heritable with a polygenic architecture. Both *P*.* papatasi* and *L*.* longipalpis* had alleles for survival that resided within many genes throughout their genomes. The implications for resistance conferred by many alleles, as well as inferences made about the utility of laboratory insecticide resistance association studies compared to field observations, are discussed.

## 1 INTRODUCTION

When populations occupy stressful environments or are faced with novel challenges, extinction can result unless populations rapidly adapt (Bell, & Gonzalez, 2009; Gomulkiewicz, & Holt, 1995; Orr, & Unckless, 2014). Such evolutionary rescue can prevent species loss from global change, but can also hamper attempts to eradicate pests and pathogens. Populations can adapt to such challenges from standing genetic variation or from new mutations, both of which can have various levels of genetic complexity from single genes of large effect to many genes with individually smaller effects (Barrett & Schluter, 2008; Chevinm, Lande, et al., 2010; Chevin, Martin, et al., 2010; Orr, 2005). Standing genetic variation is expected to contribute disproportionately to the early stages of rapid adaptation, especially in multicellular Eukaryotes (e.g Barrett & Schluter, 2008; Burke et al., 2010; Messer, & Petrov, 2013; Rêgo et al., 2019). As such, a better understanding of standing genetic variation for survival and fecundity in novel, stressful environments is critical for predicting a specie's response and persistence under such conditions.

Human activities have caused novel selective pressures and resulted in species decline (Bell & Collins, 2008). One of the best examples of human‐induced selective pressures is insecticide exposure and the evolution of insecticide resistance. Since the 1940s, synthetic insecticides have been used successfully to reduce disease transmission from arthropod vectors, but today resistance has been documented in the most important vectors including mosquitoes, black flies, triatomines, lice, fleas, and sand flies to the insecticides that are used in vector control campaigns (Alexander & Maroli, 2003; Hemingway & Ranson, 2000; Palumbi, 2001; Rivero et al., 2010). The continued application of insecticides, both in designed application and indiscriminately causing repeated exposures, and subsequent evolution of resistance have dampened enthusiasm for disease eradication in favor of sustained control (Hemingway et al., 2002; Nauen, 2007). Despite this, insecticides remain a reliable tool for controlling vectors, but their utility is eroding as fewer insecticides remain viable for control (Hemingway & Ranson, 2000; Hemingway et al., 2016; Kelly‐Hope et al., 2008). New classes of insecticides have only recently been developed and are being evaluated for efficacy and implementation (Agumba et al., 2019). Integrated approaches are needed to possibly move away from total reliance on insecticides (Kelly‐Hope et al., 2008).

Phlebotomine sand flies (Diptera: Psychodidae) are vectors that transmit *Leishmania* protozoans that cause leishmaniasis in humans, a disfiguring, stigmatizing, and lethal disease which causes tens of thousands of deaths each year worldwide (Alvar et al., 2012; Hotez, 2008; World Health Organization [WHO], 2010; WHO, 2013). Only females in the genera *Phlebotomus* and *Lutzomyia* are the competent, putative vectors of these parasites (Akhoundi et al., 2016). *Phlebotomus papatasi* (Scopoli) and *Lutzomyia longipalpis* (Lutz and Neiva) are two important vectors of *Leishmania* protozoans to people in the Old World and New World, respectively (Belo et al., 2013; Maroli et al., 2013). Sand flies, including *P*.* papatasi* and *L*.* longipalpis*, remain for the most part, susceptible to insecticides (Coleman et al., 2011). There is, though, increasing evidence of insecticide resistance in the Middle East, southern Asia, and South America (Alexander et al., 2009; Dinesh et al., 2010; Faraj et al., 2012; Hassan et al., 2012, 2015; Karakus et al., 2017; Khan et al., 2015; Saeidi et al., 2012; Surendran et al., 2005). Despite the recent findings of resistance in sand fly populations around the world, there is little knowledge about the genetic and molecular mechanisms of resistance in these populations. An understanding of these mechanisms will be crucial for the success of sand fly control programs to reduce the leishmaniasis burden without exacerbating resistance. Vector control programs based on known mechanisms of insecticide resistance in sand fly populations will have a starting point to make informed, effective control decisions about using alternative insecticides or using other integrated control methods (Alexander et al., 2009; Alexander & Maroli, 2003; Faraj et al., 2012; Surendran et al., 2005).

Conventional insecticide resistance testing often focuses primarily on the mechanisms of target‐site insensitivity and metabolic detoxification (ffrench‐Constant et al., 2004; Hemingway et al., 2004; Nauen, 2007). However, resistance is likely more complicated. Many genes with different mechanisms can collectively contribute to the resistance phenotype (David et al., 2005; Vontas et al., 2005, 2007). For example, whole‐genome sequencing also has revealed high complexity of copy number variation at insecticide resistance loci in malaria mosquitoes (Lucas et al., 2019). More robust methods are now needed to scan the sand fly genome for genetic markers associated with insecticide exposure survival.

The goal of this study is to quantify standing genetic variation for survival following insecticide exposure in laboratory populations of insecticide‐susceptible *P*.* papatasi* and *L*.* longipalpis*. To that end, we used genotype‐by‐sequencing (GBS) and multi‐locus genome‐wide association methods to quantify standing genetic variation for resistance to two insecticides (malathion and permethrin) and identify genetic loci associated with insecticide resistance (Comeault et al., 2014, 2015; Romay et al., 2013). While such methods result in only a modest density of genetic markers relative to whole‐genome sequencing, they provide a cost‐effective approach to sequence a sufficient number of individuals for genetic mapping feasible in non‐model systems. We discuss the strengths and limitations of such approaches for mapping in more detail in light of our specific results in the discussion.

## 2 MATERIALS AND METHODS

### 2.1 Sand fly colonies

Laboratory colonies of insecticide‐susceptible *P*.* papatasi* and *L*.* longipalpis* were maintained at Utah State University (USU) in Logan, UT, USA. Both species were derived from 30‐year established colonies maintained at the Walter Reed Army Institute of Research (WRAIR; Silver Spring, MD) that had been originally collected from the country Jordan and Jacobina, Brazil. All life stages were maintained and reared according to Denlinger et al. (2015) and Denlinger, Li, et al. (2016).

### 2.2 Insecticide exposure and survival phenotype scoring

Adult male and un‐blood‐fed female *P*.* papatasi* and *L*.* longipalpis* were exposed to a lethal concentration (LC) of either permethrin (*n* = 192 per species) or malathion (*n* = 192 per species), which can each cause some percent mortality. Using a modified CDC bottle bioassay protocol (Denlinger et al., 2015), *P*.* papatasi* were exposed to 50 μg/ml permethrin (LC51) and 25 μg/ml malathion (LC57), while *L*.* longipalpis* were exposed to 25 μg/ml permethrin (LC63) and 10 μg/ml malathion (LC68). These doses were previously validated for artificial selection of insecticide survival (D. S. Denlinger et al., unpublished data).

Following insecticide exposure, all sand flies were captured via mechanical aspiration and released into 1‐pint cardboard containers with a mesh top onto which a cotton ball saturated with 30% sugar‐water was placed and served as an energy/water source. The containers were kept in the same growth chamber as the insecticide‐susceptible colonies. Sand flies were held in these containers for 24‐h when mortality was observed as a complete cessation of movement (Denlinger et al., 2015; Perea et al., 2009). Here, phenotypes for insecticide resistance were assigned as a binary score of surviving (1) or dying from exposure (0). For *P*.* papatasi*, permethrin exposure resulted in 64.6% survival (*n* = 128 alive, *n* = 64 dead) while malathion led to 23.4% survival (*n* = 45 alive, *n* = 147 dead; Table 1). For *L*.* longipalpis*, permethrin exposure resulted in 65.1% survival (*n* = 125 alive, *n* = 67 dead) while malathion led to 34.9% survival (*n* = 67 alive, *n* = 125 dead; Table 1).

**TABLE 1:** Summary statistics for the *Phlebotomus papatasi* and *Lutzomyia longipalpis* permethrin and malathion treatments: insecticide exposure survival, number of individuals yielding DNA, number of DNA sequences obtained and the percent that aligned to the reference *P*.* papatasi* and *L*.* longipalpis* genomes from VectorBase, the average genome coverage, the number of variants called for each species (combining both treatments), and the minor allele frequency (MAF) correlation between survivors and dead individuals in each treatment

Treatment	Insecticide exposure survival (%)	Num. individuals yielding DNA	Total num. DNA sequences (% aligned to reference)	Avg. coverage	Num. variants	MAF correlation (*r*)
*Phlebotomus papatasi* ‐ Permethrin	128/192 (66.7)	187	80,516,505 (64)	6×	38,657	0.985
*Phlebotomus papatasi* ‐ Malathion	45/192 (23.4)	192	221,625,299 (50.4)	6×		0.987
*Lutzomyia longipalpis* ‐ Permethrin	130/192 (67.8)	182	207,072,345 (37.7)	16×	18,856	0.981
*Lutzomyia longipalpis* ‐ Malathion	96/192 (50.0)	153	75,785,403 (45.0)	16×		0.986

### 2.3 DNA isolation and library preparation

Reduced complexity, double‐digest restriction‐site DNA libraries were prepared for *P*.* papatasi* and *L*.* longipalpis* exposed to permethrin (*n* = 192 per species) or malathion (*n* = 192 per species) treatments. For each library, total DNA was isolated from individual sand flies using Qiagen's DNeasy 96 Blood & Tissue Kit (Qiagen Inc.). Following Parchman et al. (2012) with modifications from Gompert et al. (2014), DNA was digested with the restriction enzymes EcoRI and MseI (NEB, Inc.) and adaptor oligonucleotides were ligated onto the digested DNA fragments. The adaptor oligonucleotides included an Illumina adaptor and unique 8–10 bp identification sequences or barcodes for individual sand fly recognition. Fragment libraries were PCR‐amplified, pooled together, and fragments between 200 and 300 bp were selected and purified using a Blue Pippin (Sage Science) at the USU Center for Integrated Biosystems (Logan, UT, USA). All DNA libraries (*n* = 4) were sequenced at the University of Texas Genomic Sequencing and Analysis Facility (Austin, TX, USA). Libraries were sequenced on an Illumina HiSeq 2500 (permethrin‐exposed *P*.* papatasi*) and an Illumina HiSeq 4000 (malathion‐exposed *P*.* papatasi*, malathion‐ and permethrin‐exposed *L*.* longipalpis*). In total, we obtained ~585 million single‐end 100 bp DNA sequences (Table 1).

### 2.4 DNA sequence alignment and variant calling

Custom perl scripts were used to first demultiplex pooled DNA sequences, wherein identifier barcodes served to assign DNA sequences to individual sand flies (Gompert et al., 2012). Reference genomes were obtained for *L*.* longipalpis* (*Lutzomyia longipalpis jacobina* = LlonJ11; 154.2 Mbp) and *P*.* papatasi* (*Phlebotomus papatasi Israel* = PpapI1; 363.8 Mbp) from the center for invertebrate vectors of human pathogens (VectorBase; Giraldo‐Calderón et al., 2015). We used the “aln” algorithm from “bwa” (version 0.7.5; Li & Durbin, 2009) to align the DNA sequences to the *L*.* longipalpis* or *P*.* papatasi* reference genome (Table 1). We allowed for a maximum of four nucleotide differences, no more than two mismatches in a 20 bp seed, and a quality threshold for read trimming set to 10. Along with these parameters, only reads with a single best match were aligned. A small number of sand flies with few aligned sequences were removed before subsequent analyses. Sequence coverage was 6× for *P*.* papatasi* compared to 16× for *L*.* longipalpis*, consistent with the larger genome size of *P*.* papatasi* than *L*.* longipalpis* (Table 1).

Using “samtools” and “bcftools” (version 0.1.19; Li et al., 2009), sequence alignments were sorted and indexed for variant calling. Treatment groups of each sand fly species were combined for this process. As recommended for Illumina HiSeq data, coefficients to cap mapping quality and the number of reads per position were set to 50. Bases with a quality score below 15 and reads with a mapping quality below 10 were ignored. The prior for *θ* was set to 0.001, and only single nucleotide variants (SNVs) where the posterior probability of an invariant nucleotide was below 0.01 were retained (Li, 2011). Each SNV set was filtered based on a 128‐read minimum for overall coverage, a four‐read minimum for the non‐reference allele, a minimum mapping quality of 30, and a maximum of 20% of individuals with missing data. Our filtering criteria were selected to ensure sufficient coverage to estimate allele frequencies while accounting for uncertainty in genotype (Buerkle & Gompert, 2013), and while also avoiding locus drop‐in and drop‐out (that is, where mutations create or remove DNA sequence motifs cut by the restriction enzymes). Final sets of 38,657 and 18,856 SNVs (~1 SNV per 10 kpb for each species) were retained for *P*.* papatasi* and *L*.* longipalpis*, respectively (Table 1).

### 2.5 Estimating genotypes, allele frequencies, and linkage disequilibrium

We estimated allele frequencies for each species and insecticide treatment. Maximum likelihood allele frequency estimates were obtained using an expectation‐maximization algorithm that accounts for uncertainty in genotypes (Gompert et al., 2014; Li, 2011). Relative to methods that rely on first calling genotypes, this approach has the advantage of allowing for the inclusion of individuals with a range of sequence coverage and weighting their contributions to the allele frequency estimates by the information carried in their sequence data (Buerkle & Gompert, 2013).

Genotype estimates are required for association mapping. Thus, we next used a Bayesian approach to estimate genotypes for each SNP and individual. Our empirical Bayesian approach uses the allele frequency estimates to define prior probabilities for genotypes, such that Pr(*g* = 0) = (1 − *p*)^2^, Pr(*g* = 1) = 2*p*(1 − *p*) and Pr(*g* = 2) = *p* ^2^ where *g* denotes the counts of, for example, the non‐reference allele (0, 1 or 2 in diploids) and *p* denotes the corresponding allele frequency. Posterior probabilities were then obtained according to Bayes rule as Pr(*g*| D, *p*) = [Pr(D|*g*) Pr(*g*)]/Pr(D), where Pr(D|*g*) defines the likelihood of the genotype given the sequence data and quality scores as calculated by samtools and bcftools. We then obtained point estimates (posterior means) of genotypes as Pr(*g* = 0|D,*p*)*0 + Pr(*g* = 1|D,*p*)*1 + Pr(*g* = 2|D,*p*)*2. This results in genotype estimates that take on values between 0 and 2 (copies of the non‐reference allele) but that are not constrained to be integer valued).

Pairwise linkage disequilibrium (LD) was calculated in each species from our genotype estimates using the “geno‐r2” function “vcftools” (version 0.1.15; Danecek et al., 2011). Specifically, we measured LD as the squared correlation between genotypes at pairs of SNPs and computed LD for all pairs of SNPs in 100 kb windows.

### 2.6 Genome‐wide association mapping

Binary Bayesian sparse linear mixed models (BSLMMs) were fit with “gemma” (version 0.98; Zhou et al., 2013) to estimate genetic contributions to variation in insecticide survival and to identify SNVs with this phenotypic variation. Here, survival outcomes were modeled as a function of a polygenic term (denoted *u*) and a vector of the potential measurable SNV effects (denoted β) (Zhou et al., 2013). A Markov chain Monte Carlo (MCMC) algorithm with variable selection was used to infer posterior inclusion probabilities (PIPs) for SNVs with a non‐zero measurable effect on insecticide susceptibility, and model‐average point estimates (MAPEs) were derived from those PIPs (Zhou et al., 2013). The polygenic term in each BSLMM represents expected deviations from a phenotypic mean based on all SNVs while accounting for phenotypic covariance that arise between sand flies due to relatedness or genetic similarity (i.e., observed kinship; Zhou et al., 2013). Relatedness was also considered when estimating individual SNV effects (β) and their PIPs with kinship matrices.

Parameters for estimating genetic architecture were derived from the hierarchical structure of the BSLMM (Guan & Stephens, 2011; Lucas et al., 2018; Zhou et al., 2013). Altogether, the parameters indicate the proportion of the phenotypic variance explained (PVE) by additive genetic effects (based on β and the polygenic term), the proportion of PVE explained by measurable‐effect SNVs (PGE) or those implicated by LD (β alone), and the number of SNVs with effects that explain phenotypic variance (*n*‐γ).

Thirty independent MCMC chains were run for binary BSLMMs, wherein a probit link function was used to connect the binary response (survival outcome) to a latent quantitative risk variable. MCMC chains included 100,000 burn‐in steps, 1 million sampling steps, and a thinning interval of 10. We assessed convergence to the posterior distribution by calculating the Gelman–Rubin potential scale reduction diagnostic for PVE, PGE and *n*‐γ in R with the “CODA” package (version 0.19.3; Plummer et al., 2006; R Core Team, 2013); values of this statistic for were generally less than 1.1 consistent with convergence. To reduce bias in estimation, inferences were carried out using the combined values from all iterations across chains (Cowles & Carlin, 1996).

### 2.7 Insecticide survival predictions

We used five‐fold cross‐validation to evaluate the predictive power of the genome‐wide association mapping models. To do this, we refit the BSLMM model five times for each data set (species and insecticide treatment). In each case, we used a random 80% of the observations as a training set to fit the model and the other 20% to evaluate the model. We fit the BSLMM models via MCMC with 100,000 steps as a burn‐in, followed by 1 million sampling steps with a thinning interval of 10. The fit model was used to predict the survival phenotype of the test individuals, that is to obtain genomic‐estimated breeding values for each of the test individuals based on the additive effects of genes were captured by both β and *u* in the BSLMMs (Gompert et al., 2019; Lucas et al., 2018). We used the full set of predictions across the five‐fold cross‐validation sets to assess predictive performance. This was done using the R package “ROCR” (version 1.0.7; Sing et al., 2005); receiver operator characteristic (ROC) curves were constructed to interpret the area under the curve (AUC) and determine the predictive power in correctly classifying survival outcomes.

### 2.8 Variant effect predictions

We used the Ensembl Variation Effect Predictor on VectorBase to characterize the genomic context and possible consequences of each SNV in the data set, that is to classify SNV based on their effect if in exons (e.g., synonymous, missense, etc.) or genomic context if not (e.g., intron, 3′ UTR, 5′ UTR, intergenic, etc.) (Giraldo‐Calderón et al., 2015; McLaren et al., 2010, 2016). We then summarized the annotations for the 100 SNVs most associated with survival in each treatment for each species and used randomization tests (1000 randomizations each) to determine whether any category was over‐represented relative to null expectations.

## 3 RESULTS

### 3.1 Genetic variation

**FIGURE 1:**
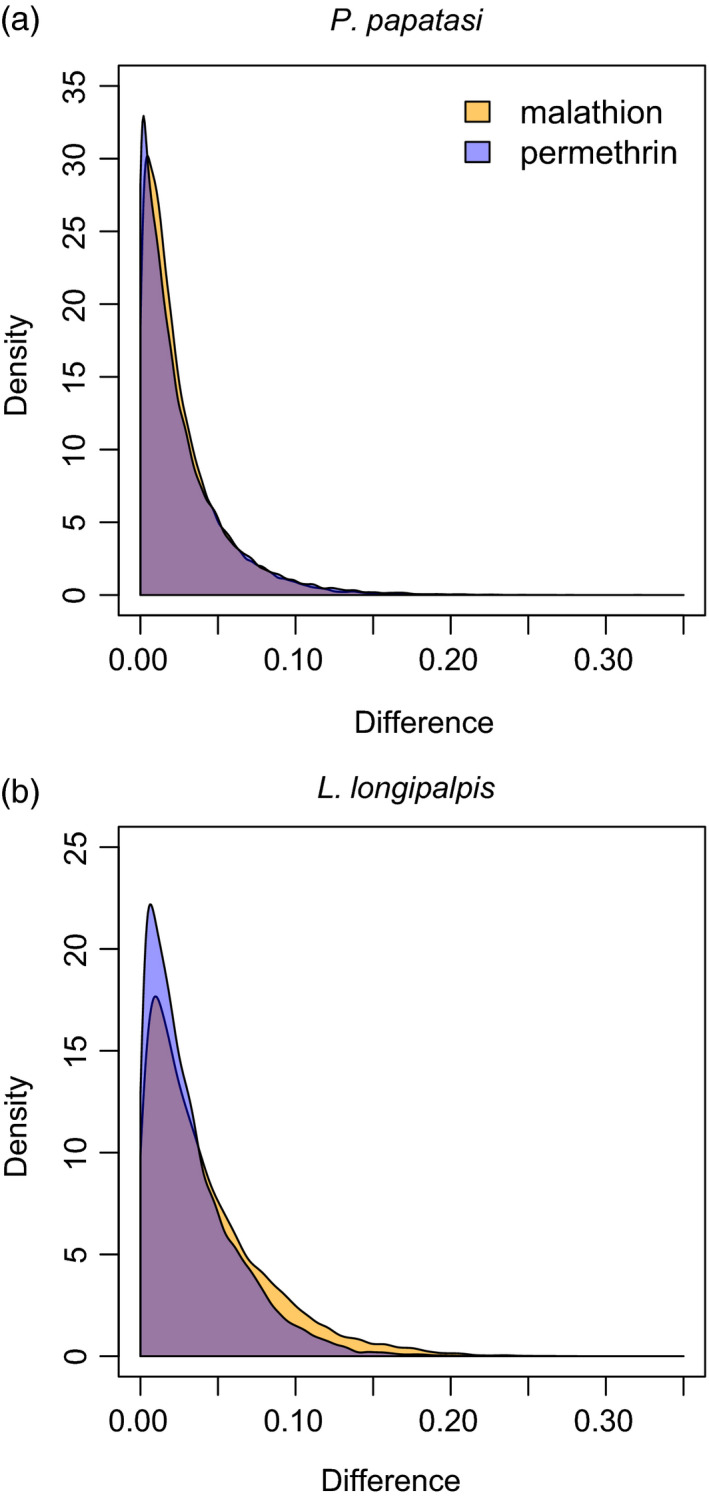
Density plots show the distribution of allele frequency differences between surviving and dead sand flies for each treatment (permethrin or malathion) for *Phlebotomus papatasi* (a) and *Lutzomyia longipalpis* (b)

As expected, allele frequencies were highly correlated between surviving and dead sand flies for each species and treatment (Table 1, Figure S1). Average allele frequency differences (i.e., the mean, absolute difference in the frequency of each allele) between surviving and dead flies were 0.042 (malathion) and 0.033 (permethrin) in *L*.* longipalpis* and ~0.025 (both treatments) in *P*.* papatasi* (Figure 1). Nonetheless, change for some SNVs was much higher, with maximum values of 0.23–0.32 across species and insecticide treatments. Also as expected, greater allele frequency differences between surviving and dead flies was seen for SNVs with higher minor allele frequencies (i.e., more genetic variation; Pearson correlations between 0.36 and 0.49, all *p* < 0.001).

**FIGURE 2:**
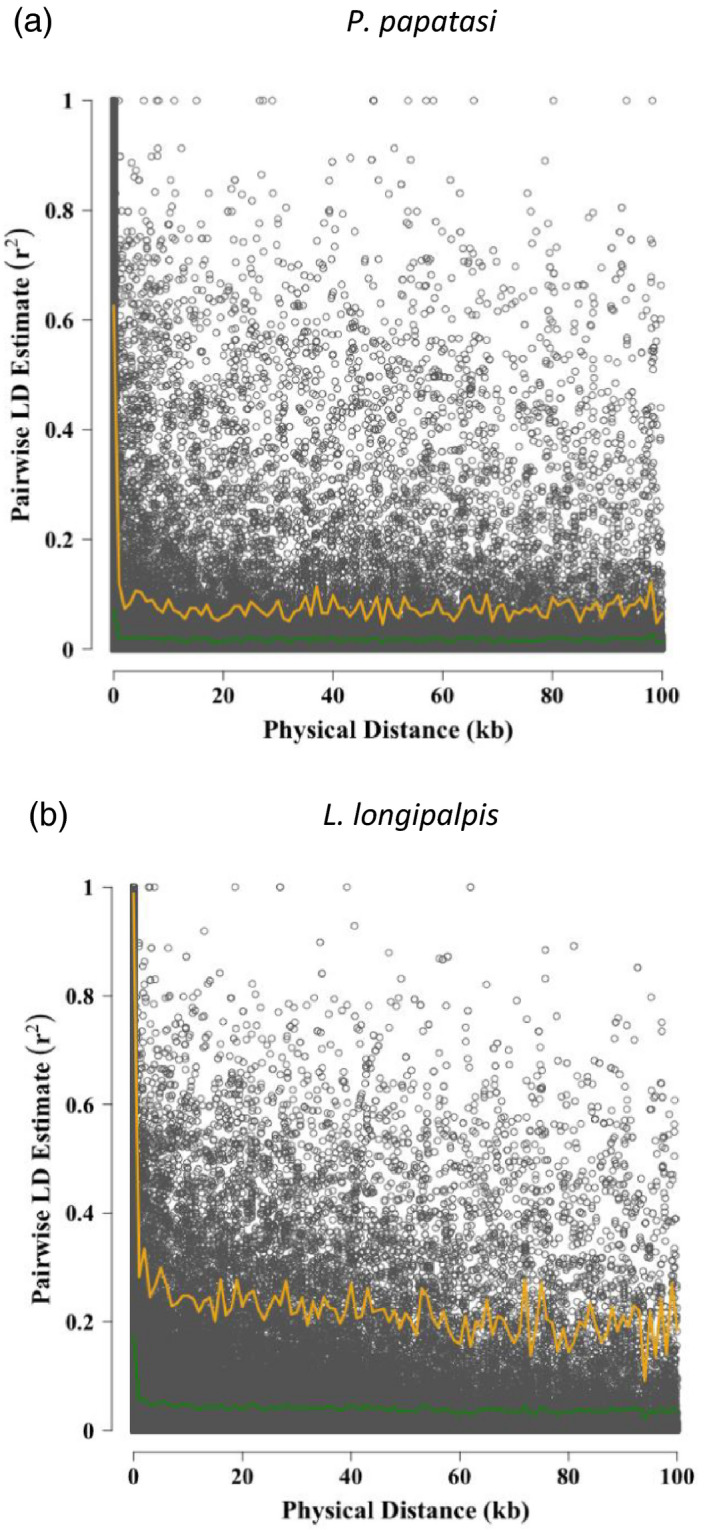
Linkage disequilibrium (LD) decay as a function of physical distance across the genomes of *Phlebotomus papatasi* (a) and *Lutzomyia longipalpis* (b). The orange lines denote the 95% quantile of LD, and the green lines denote the mean LD

Linkage disequilibrium decayed with physical genomic distances in both *P*.* papatasi* and *L*.* longipalpis* (Figure 2). Nonetheless, non‐trivial LD persisted at a sufficient distance for the SNV markers to likely exhibit LD with at least a reasonable proportion of causal variants. In particular, with a marker density of ~1 SNV per 10 kb, we would expect most causal variants to be within 5 kb of at least one SNV maker. At the scale of 5 kb, mean LD measured by r^2^ was 0.021 in *P*.* papatasi* (maximum = 1.0) and 0.047 in *L*.* longipalpis* (maximum = 0.80).

### 3.2 Genome‐wide association mapping

Point estimates of the proportion of variation in survival explained by additive genetic effects (PVE) ranged from 14.7% for *P*.* papatasi* exposed to malathion to 90.1% for *L*.* longipalpis* exposed to malathion (Table 2). However, these estimates were associated with considerable uncertainty (Table 2). Moreover, with the exception of *P*.* papatasi* exposed to permethrin, we lacked sufficient data for precise estimates of the proportion of the PVE attributable to individual genetic variants with measurable effects (PGE) versus near‐infinitesimal effects. Estimates of the number of causal variants with measurable effects (*n*‐γ) were lower in *P*.* papatasi* than *L*.* longipalpis* for both insecticides (Table 2).

**TABLE 2:** Phenotypic variance explained (PVE), PGE, and *n*‐ γ posterior medians and 95% credible intervals (CrI) for the *Phlebotomus papatasi* and *Lutzomyia longipalpis* permethrin and malathion treatments

Treatment	PVE (CrI)	PGE (CrI)	*n*‐γ (CrI)
*Phlebotomus papatasi* ‐ Permethrin	62% (26.7%–99.9%)	69.7% (31.0%–100%)	7 (0–60)
*Phlebotomus papatasi* ‐ Malathion	14.7% (0.0001%–53.6%)	36.4% (0%–93.0%)	15 (0–217)
*Lutzomyia longipalpis* ‐ Permethrin	35.6% (0.001%–76.1%)	39.5% (0%–93.5%)	28 (0–243)
*Lutzomyia longipalpis* ‐ Malathion	90.1% (40.7%–99.9%)	29.8% (0%–91.6%)	58 (0–258)

**FIGURE 3:**
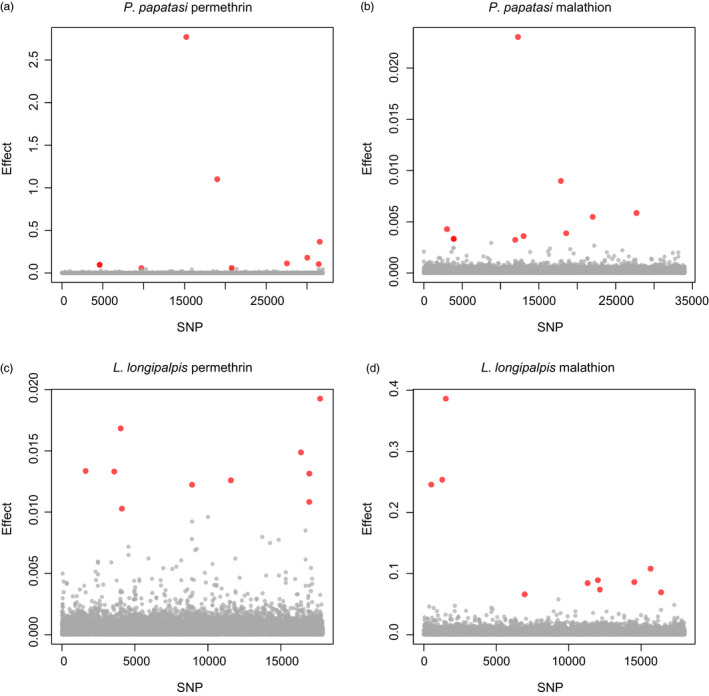
Estimated effects of each SNV on survival in the *Phlebotomus papatasi* permethrin treatment (a), *Phlebotomus papatasi* malathion treatment (b), *Lutzomyia longipalpis* permethrin treatment (c), and the *Lutzomyia longipalpis* malathion treatment (d). Points denote absolute values of model‐averaged effect estimates, that is estimates weighted by the posterior probability of a non‐zero effect. In each panel, the effects of the 10 SNVs with the largest estimates are shown in red

**FIGURE 4:**
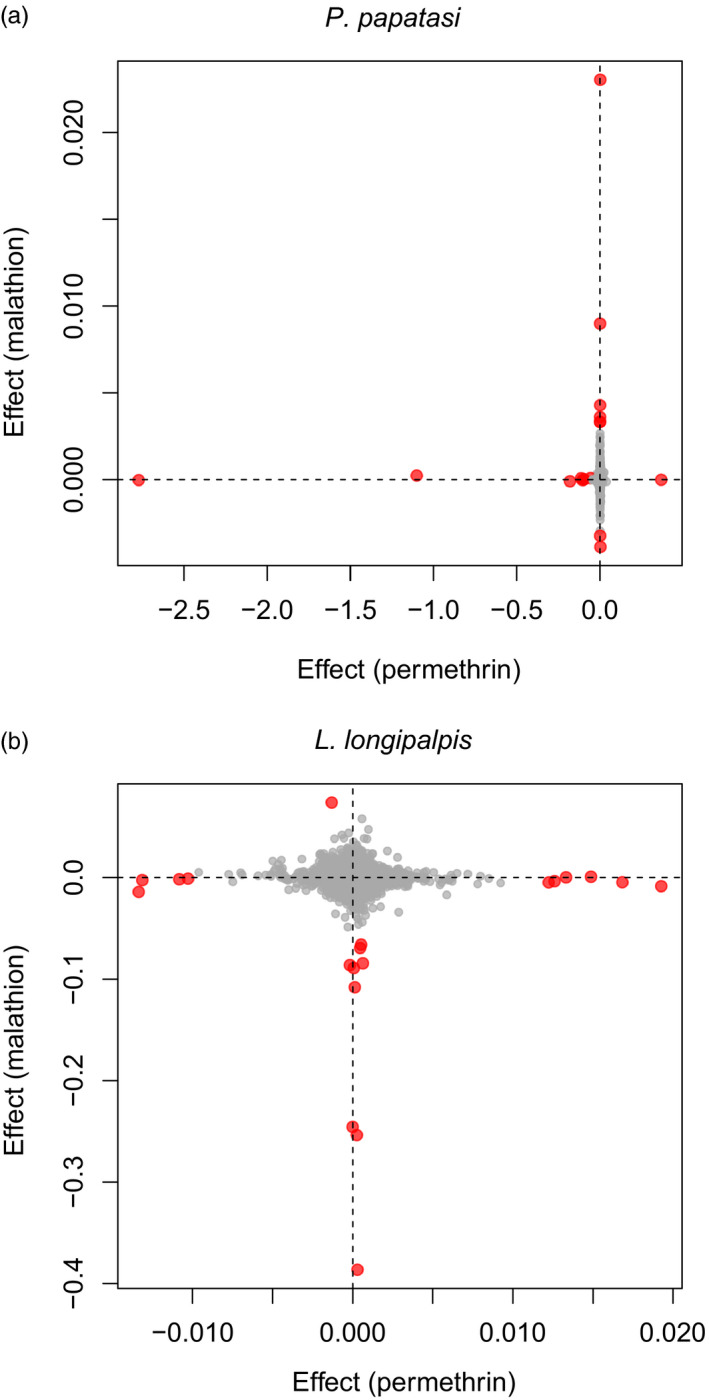
Scatterplots depict the associations between estimated SNV effects on survival in the permethrin versus malathion treatments for *Phlebotomus papatasi* (Pearson *r* = −0.001, 95% CI = −0.013 to 0.010, *p* = 0.82) (a) and *Lutzomyia longipalpis* (Pearson *r* = −0.035, 95%CI = −0.050 to −0.020, *p* < 0.001) (b). Points denote signed, model‐averaged effect estimates, that is estimates weighted by the posterior probability of a non‐zero effect. In each panel, the effects of the 10 SNVs with the largest estimates are shown in red. Dashed lines in each panel denote no effect

Consistent with this, several SNVs had modest to large PIPs for *P*.* papatasi* exposed to permethrin (six SNVs with PIPs >0.05 and two with PIPs >0.4) and correspondingly large model‐averaged effect estimates (Figure 3a), whereas for the other treatment and species combinations, no SNVs had PIPs >0.05 (Figure 3b–d). In each species, model‐averaged SNV effect estimates were mostly independent for the two insecticide treatments. In other words, SNVs most strongly associated with survival in one insecticide treatment (e.g., permethrin), were not associated with survival in the other treatment (e.g., malathion) (Figure 4). Although there was very little LD detected among large‐effect SNVs for malathion survival, moderate LD was present for SNVs with high effects on permethrin survival (Figure S2).

### 3.3 Insecticide survival predictions

**FIGURE 5:**
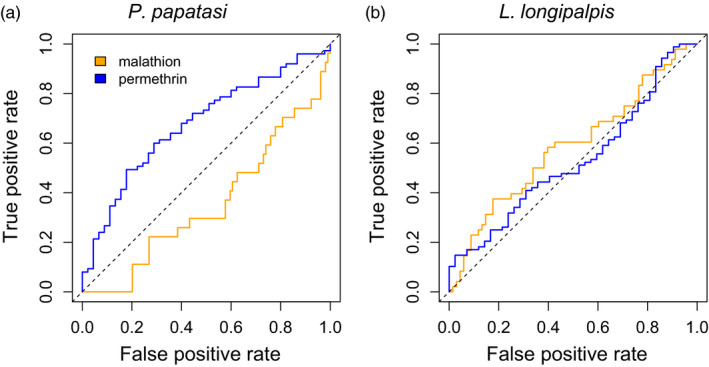
Predictive power of the genome‐wide association models based on receiver operating characteristic (ROC) curves. ROC curves are shown for *Phlebotomus papatasi* survival when exposed to malathion (area under the ROC curve [AUC] =0.36) and permethrin (AUC = 0.68) (a) and *Lutzomyia longipalpis* when exposed to malathion (AUC = 0.59) and permethrin (0.53). Dashed lines show expectations for a model with no predictive power (AUC = 0.5)

Standing genetic variation in *P*.* papatasi* was moderately sufficient in predicting permethrin survival (AUC = 0.68, which denotes a 36% increase in predictive power relative null expectations of AUC = 0.5; Figure 5a), but was no better than null expectation in terms of predicting malathion survival (AUC = 0.36; Figure 5b). In *L*.* longipalpis*, we observed a small but non‐zero increase in predictive power relative to a null model for both permethrin (AUC = 0.53; Figure 5c) or malathion (AUC = 0.59, Figure 5d) exposure.

### 3.4 Variant effect predictions

**FIGURE 6:**
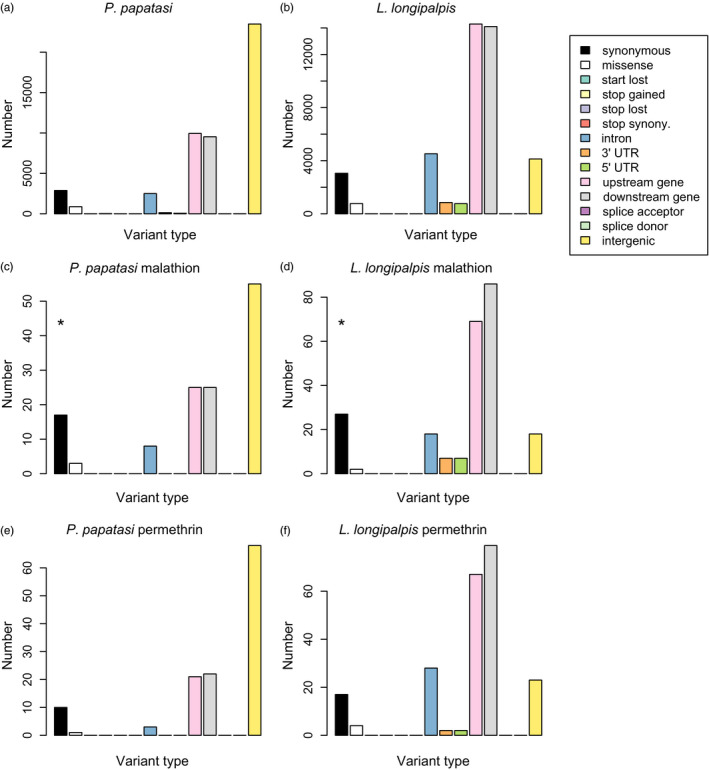
Barplots show the genetic variant type or consequences for all SNVs (a, b), and the 100 SNVs with the largest model‐averaged effects on survival with exposure to malathion (c, d) or permethrin (e, f). Annotations are based on the variant predictor tool in VectorBase. Asterisks denote categories that are significantly over‐represented among the top 100 SNVs relative to null expectations based on the full set of SNVs in each species (randomization test, 1000 randomizations, *p* < 0.05)

In both species, most SNVs occurred outside of genes (e.g., VEP categories intergenic, upstream or downstream of genes) (Figure 6a,b). Nonetheless, we detected thousands of SNVs in gene introns or coding sequences. In general, annotations for the 100 SNVs most strongly associated with survival in each insecticide treatment (i.e., 100 SNVs with the largest model‐averaged effect estimates) were consistent with null expectations based on the full set of SNVs (Figure 6c–f). The only exceptions involved an over‐representation of synonymous, genic SNVs among those associated with survival of malathion exposure in both *P*.* papatasi* and *L*.* longipalpis* (*p* = 0.002 and 0.010 from a randomization tests, respectively).

## 4 DISCUSSION

We found evidence of standing genetic variation for reduced susceptibility to permethrin and malathion in susceptible lab colonies of *P*.* papatasi* and *L*.* longipalpis*. This suggests a potential for these species to evolve resistance to these insecticides. We discuss our estimates of the genetic architecture of resistance and possible implications of our results below. But first, we highlight what we think are the most critical limitations of the current study. With the GBS approach, we only sequenced a subset of the genome. Because of this, it is possible and even likely that some causal variants were not in LD with the SNVs we sequenced (Catchen et al., 2017; Lowry et al., 2017; McKinney et al., 2017). Moreover, even where we did have SNVs in LD with causal variants, we may have underestimated their effects because these statistical associations (i.e., LD) were imperfect. Thus, we have likely missed or underestimated the effects of an unknown subset of resistance alleles. Still, given our marker density and patterns of LD, the GBS approach allowed us to sequence a sufficient number of flies to identify some genetic markers associated with resistance and the overall contribution of genetics to the trait (i.e., the PVE). Thus, we think this approach was useful, but some caution is warranted when interpreting our results. Second, our results apply specifically to these lab colonies. It is simply unclear at this time whether or to what extent the same genetic variants are segregating in nature. Still, given the fact that standing genetic variation is often shared among populations, we think it is quite likely that at least some of these same causal variants are segregating in nature.

### 4.1 Genetic architecture

In *P. papatasi*, survival to a sub‐diagnostic dose of permethrin is heritable (PVE posterior median = 62%; CrI = 26.7%–99.9%), and most of that heritability comes from SNVs with individually measurable effects for survival (PGE posterior median = 69.7%; CrI = 31.0%–100%; here, we equate PVE with narrow‐sense heritability but note that it can be an underestimate of heritability if a subset of causal variants is not in LD with our SNP loci). Moreover, we had some ability to predict whether an insecticide‐susceptible *P*.* papatasi* will survive or die from an exposure to a sub‐lethal dose of permethrin based on this polygenic model. Interestingly, survival with a sub‐lethal dose of malathion was only about a fifth as heritable as survival to a sub‐lethal dose of permethrin (posterior median = 14.7%; CrI = 0.0001%–53.6%), and different SNVs and genes were associated with survival although further associated studies are needed. Perhaps the susceptible population of *P*.* papatasi* did not already have the genetic variation to survive malathion's different mode of action from permethrin. However, it is important to note that the effectual LC that flies were exposed to was higher for malathion than for permethrin, and thus the sand flies could harbor additional standing genetic variation for survival to lower concentrations of malathion. Posterior inclusions probabilities for the highest ranking SNVs were also much lower, and not surprisingly, our power to predict survival to exposure to malathion was considerably lower too.

Phenotypic variation for survival ability in permethrin‐exposed *L*.* longipalpis* was moderately heritable (posterior median = 35.6%; CrI = 0.001%–76.1%). To some extent, the genetic underpinnings for such variation can be explained by a small number of causal variants (posterior median = 28; CrI = 0–243) with measurable effects for survival (posterior median = 39.5%; CrI = 0%–93.5%). Regardless, given the genotypes of insecticide‐susceptible *L*.* longipalpis*, there was only very slight power to predict whether survival or death will result from a sub‐lethal exposure to permethrin given their genotypes. This lack of predictive power could in part be due to the moderate levels of heritability for causal variants associated with survival. Conversely, *L*.* longipalpis* survival ability when exposed to a sub‐lethal dose of malathion is very heritable (posterior median = 90.1%; CrI = 40.7%–99.9%), but much of the genetic basis is owed to many SNVs (posterior median = 58; CrI = 0–258) with infinitesimal effects (posterior median = 29.8%; CrI = 0%–91.6%). This finding is reflected by the relatively low model average point estimates and posterior inclusion probabilities associated with candidate SNVs, as well as the low predictive power for the survival phenotype. Given the genotypes of insecticide‐susceptible *L*.* longipalpis*, and despite the significant heritability, there is only moderate predictive power whether survival or death will result from a sub‐lethal exposure to malathion.

### 4.2 Gene associations

Intergenic variants and variants associated with genes were among the top five highest ranking SNVs in all four treatment groups. The variants associated with genes were found in genes or upstream or downstream of them. Some genes do not yet have an annotated function in the sand fly genomes. The genes that are annotated have a diverse range of metabolic and biochemical functions (Tables S1–S4). We must be cautious, though, in our inferences. Despite being able to analyze tens of thousands of variants, only a small portion of the genome is sequenced with GBS. Some of the variants we found associated with survival to an insecticide exposure may be causal; but the vast majority are likely only associated with causal variants via LD. Also, some of these genes have been associated with insecticide resistance in other vectors and agricultural pests. Even the intergenic variants could serve important biochemical functions as gene expression regulators (Elshire et al., 2011).

Serine proteases (high MAPE score in the *P*.* papatasi* malathion exposure), like acetylcholinesterases, are inhibited by organophosphates, like malathion. They are up‐ or downregulated in resistant insects (Chambers & Oppenheimer, 2004; Vontas et al., 2007) and are important for synthesis and conformation of detoxifying enzymes in the presence of organophosphates (Ahmed et al., 1998). Zinc fingers (high MAPE scores in the malathion exposure in both sand fly species) are transcriptional repressors (Kasai & Scott, 2001). In *Musca domestica*, mixed functional oxidase (MFO), a class of insecticide detoxifying enzymes, promoters bind transcription repressor genes that contain zinc finger moieties. The MFO promoters in pyrethroid‐resistant *M*.* domestica* bind the repressor genes less than in susceptible individuals because of polymorphisms in the repressor gene. This causes increased transcription of MFOs, which are able to detoxify pyrethroid insecticides (Gao & Scott, 2006; Perera et al., 2008). It is possible that the upstream variant of the zinc finger encoding gene contributes to MFO repression. Decreased MFOs can also confer resistance because they first must enzymatically activate insecticide, which they later detoxify. With fewer MFOs, there are fewer bioactivated insecticides (Scott, 1999). Perhaps variants near or within zinc fingers contribute to increased or decreased MFO expression and either can lead to insecticide resistance.

Several SNVs were found that are associated with proteins in the *L*.* longipalpis* malathion‐exposure treatment (Table S4). A SNV was found to be associated with a protein containing a disulfide isomerase function. GSTs in insects are known to alter isomerase activity (Sheehan et al., 2001). In the same treatment, microtubule associated protein RP/EB were upregulated found in lambda‐cyhalothrin resistant *Aphis glycines*. Microtubule associated proteins interact with postsynaptic proteins in the nervous system. They could help stabilize dendrites to normalize nerve function when malathion disrupts synaptic transmission by inhibiting acetylcholinesterase (Lepicard et al., 2014). Intra‐flagellar transport proteins were less abundant in imidacloprid‐resistant *Myzus persicae* (Meng et al., 2014). Glycosyltransferases are detoxification enzymes, and overexpression of some uridine diphosphate‐glycosyltransferases has been shown to confer resistance in lepidopteran agricultural pests (Li et al., 2016). Lastly, a SNV was found associated with a gene that transcribes a protein with an alpha/beta hydrolase fold activity. Carboxylesterase and cholinesterase enzymes, such as acetylcholinesterase, evolved from a core alpha/beta hydrolase, and these enzymes frequently confer insecticide resistance (Hotelier et al., 2010).

### 4.3 Standing genetic variation and adaptation

Despite more genetically homogenous laboratory populations of sand flies (Lanzaro et al., 1998; Mukhopadhyay et al., 1997, 1998, 2001), insecticide exposure survival is a known heritable trait and can lead to resistance (Feyereisen, 1995; Hemingway et al., 2002; Rivero et al., 2010). In theory, alleles for survival will increase in frequency toward fixation with continued selection, disseminate throughout the population, and result in greater population survival over the course of continued exposure (Xu et al., 2012). The rate of evolution in a population depends on multiple factors, including the initial allele frequency (Roush & McKenzie, 1987). The insecticide‐susceptible colonies used in this experiment were derived from 30‐year inbred populations that were most likely homozygous for many loci and maybe during that time emergent pre‐adaptive alleles were removed through purifying selection and/or through stabilizing selection. Despite evidence of sufficient standing genetic variation for selection to act upon, this variation could have been very little.

Polygenic insecticide resistance under laboratory conditions has been studied theoretically and empirically (David et al., 2005; ffrench‐Constant, 2013; ffrench‐Constant et al., 2004; McKenzie et al., 1992). Selection for resistance in a laboratory population falls within the phenotypic distribution of the susceptible population, often below the LC_100_ for an insecticide (ffrench‐Constant et al., 2004; Oakeshott et al., 2013; Roush & McKenzie, 1987). This selection process is conducted to allow survivors for subsequent generations. In doing so, existing, common variation is selected for, which produces polygenic resistance. Because of the homogeneity of laboratory populations, very low initial frequency of resistance alleles, the high fitness costs of those resistance alleles, and the weakness of the selection process, the evolution of resistance from major‐effect alleles is potentially unlikely (Lande, 1983; McKenzie et al., 1992). Even a LC_90_ of an insecticide has the potential to produce polygenic resistance (McKenzie & Batterham, 1994). Our lineages are being exposed to an approximate LC_50_ of permethrin and malathion, so it is certainly expected that we will find evidence of polygenic resistance. Monogenic resistance can be successfully selected for in the laboratory if selection concentration is set above the LC_100_ of an insecticide (McKenzie & Batterham, 1998). With diagnostic doses for many insecticides for sand flies recently described (Denlinger, Creswell, et al., 2016), selection for major‐effect alleles is possible in the future.

Resistance selection in field populations is much greater (above the LC_100_ for an insecticide) and can be outside of the phenotypic range of insecticide tolerance. This can result in the rapid selection of rare, major‐effect mutations that can lead to monogenic or oligogenic resistance that present as target‐site insensitivity, metabolic detoxification, or both epistatically (Edi et al., 2014; ffrench‐Constant et al., 2004; Hardstone et al., 2009; McKenzie & Batterham, 1998; Saavedra‐Rodriguez et al., 2008; Whitten et al., 1980). Here, large sizes of field populations act as a source of rare mutations, whereas the small population sizes of inbred individuals in a laboratory population only lead to an accumulation of small effect‐size mutations (ffrench‐Constant, 2013; McKenzie et al., 1992). It is the heterogeneity of field populations that allows for rare variants to exist (Groeters & Tabashnik, 2000). Interestingly, rare variants may precede the selection for resistance. For example, In Australia, mutations for organophosphate resistance in *Lucilia* blow flies predated the use of malathion. Examples of standing genetic variation of resistance alleles in field populations, prior to insecticide use, demonstrate that these alleles are under balancing selection and do not carry a high enough fitness cost (ffrench‐Constant, 2007). Alleles already present in populations are known to quickly increase in frequency from human‐induced evolution (Messer et al., 2016). This may be why resistance has evolved very rapidly when insecticides are first introduced as a control method (Hemingway & Ranson, 2000).

Laboratory strains initiated from field populations with monogenic resistance may not always evolve monogenic resistance because of the factors associated with polygenic resistance selection (Groeters & Tabashnik, 2000; Kasai et al., 2014; Zhu et al., 2013). This may be why Fawaz et al., (2016) did not find target‐site insensitivity mutations in their laboratory colony initiated from Egyptian *P*.* papatasi*. Even so, resistance in the field may be more polygenic than initially perceived, and this could be due to fitness costs and pleiotropy from major‐effect mutations. Microarrays have found many genes with various functions involved in resistance, more than could be found by simply testing for known resistance mechanisms including target‐site insensitivity and metabolic detoxification (Djouaka et al., 2008; Pedra et al., 2004; Vontas et al., 2005, 2007). These findings demonstrate that insecticide resistance, in both the field and laboratory, is a complx phenotype that combines major‐effect changes (target‐site insensitivity and metabolic detoxification) and many other alleles that are beginning to be discovered and understood.

### 4.4 Resistance control implications

Despite the theoretical work of understanding insecticide resistance in laboratory populations, it behooves insect vector management programs to be cautious about proposing management strategies based only on what has been observed in artificial‐selection experiments, as these results do not always empirically verify what is observed in the field (ffrench‐Constant, 2013). Even within different laboratory colonies of the same species or population, polygenic resistance can be different (Daborn et al., 2002; Dapkus & Merrell, 1977; ffrench‐Constant, 2013). Nevertheless, the importance of artificially selecting for resistance should not be underestimated because of the ability to predict variants of resistance mechanisms for new insecticides to be used in the field (McKenzie & Batterham, 1998).

We found that selecting for insecticide exposure survival in laboratory colonies of sand flies is possible but challenging. There is sufficient standing genetic variation in our colonies for polygenic resistance mechanisms. Polygenic resistance is not frequently found in field populations of insects because of greater selection pressure and larger pools of genetic diversity, but it is possible (Groeters & Tabashnik, 2000; Raymond & Marquine, 1994). Polygenic insecticide resistance found in the field is maintained by low mutation rates and minimal migration, both of which are a source of new alleles for monogenic resistance (Raymond & Marquine, 1994; Zhu et al., 2013).

A question that remains is whether polygenic resistance is likely in field populations of sand flies. Sand flies are weak fliers, distribute poorly, and are vagile, which together can lead to small, genetically structured populations (Belen et al., 2011; Doha et al., 1991; Hamarsheh et al., 2007; Khalid et al., 2012; Morrison et al., 1993; Orshan et al., 2016). The weaker effect of selection in smaller populations, and the stronger effect of drift, could dilute resistant alleles should they arise through mutation (Lanfear et al., 2014). Additionally, smaller populations are less likely to be rescued and more likely to go extinct (Willi et al., 2006), but this is not always true (Ferriere & Legendre, 2012). Compound these factors with little gene flow from poor migration, or with gene flow from susceptible sand flies that were unexposed to insecticide due to inadequate insecticide coverage in the environment, and susceptible alleles could remain commonplace in a population. These maladapted alleles, under insecticide selection pressure, could build up a migration load should there be migration (Bolnik & Nosil, 2007). From a control standpoint, these features could be an exploitable opportunity for a failure of evolutionary rescue that may not be seen in other insect vectors. Rapid evolutionary adaptation may not be realistic in these fragmented populations in nature because of potentially little standing genetic variation, and they would be susceptible to stochastic population decline and extinction with the relative inability for adaptation to save them (Gonzalez et al., 2013). Additionally, our findings that the SNVs associated with survival to permethrin and malathion are mostly independent suggests that cross‐resistance in sand flies to multiple insecticide classes may require many SNVs and/or mechanisms. Alternative classes of insecticides would remain viable in the presence of resistance, which would be advantageous for sand fly control programs.

For our laboratory populations, predictions, not assumptions and conclusions, should be made about the mechanisms of insecticide resistance in field populations (Mukhopadhyay et al., 1997). The results from this experiment should serve as a model, not a standard or representative of sand flies in the field. More research of survival and resistance mechanisms using GBS needs to be investigated in natural populations and incorporated into effective integrated vector management programs. GBS's utility in scanning entire genomes of vectors for markers associated with insecticide exposure survival, in both field and laboratory populations, should be incorporated into studies examining the genetic mechanisms of insecticide resistance. GBS will enhance research that examines insecticide use, refuge populations, and gene flow for when insecticide coverage for vectors is uneven, heritability and dominance levels of resistance, fitness costs, and the dynamics of polygenic resistance becoming monogenic resistance (Mallet, 1989; McKenzie et al., 1992; Neve et al., 2009; Tabashnik et al., 2003).

## Supporting information

Supplementary Material

## Data Availability

Data for this study are available at the Dryad Digital Repository (https://doi.org/10.5061/dryad.9cnp5hqh3) and at NCBI SRA (Accession: PRJNA694194 ID: 694194).

## References

[eva13194-bib-0001] Agumba, S. , Gimnig, J. E. , Ogonda, L. , Ombok, M. , Kosgei, J. , Munga, S. , Guyah, B. , Omondi, S. , & Ochomo, E. (2019). Diagnostic dose determination and efficacy of chlorfenapyr and clothianidin insecticides against *Anopheles* malaria vector populations of western Kenya. Malaria Journal, 18(1), 243. 31315614 10.1186/s12936-019-2858-z PMC6637467

[eva13194-bib-0002] Ahmed, S. , Wilkins, R. M. , & Mantle, D. (1998). Comparison of proteolytic enzyme activities in adults of insecticide resistant and susceptible strains of the housefly *M. domestica* L. Insect Biochemistry and Molecular Biology, 28, 629–639. 9755473 10.1016/s0965-1748(98)00061-7

[eva13194-bib-0003] Akhoundi, M. , Kuhls, K. , Cannet, A. , Votýpka, J. , Marty, P. , Delaunay, P. , & Sereno, D. (2016). A historical overview of the classification, evolution, and dispersion of *Leishmania* parasites in sandflies. PLoS Neglected Tropical Diseases, 10, e0004349. 26937644 10.1371/journal.pntd.0004349 PMC4777430

[eva13194-bib-0004] Alexander, B. , Barros, V. C. , de Souza, S. F. , Barros, S. S. , Teodoro, L. P. , Soares, Z. R. , Gontijo, N. F. , & Reithinger, R. (2009). Susceptibility to chemical insecticides of two Brazilian populations of the visceral leishmaniasis vector *Lutzomyia longipalpis* (Diptera: Psychodidae). Tropical Medicine and International Health, 14, 1272–1277. 19772549 10.1111/j.1365-3156.2009.02371.x

[eva13194-bib-0005] Alexander, B. , & Maroli, M. (2003). Control of phlebotomine sandflies. Medical and Veterinary Entomology, 17, 1–18. 12680919 10.1046/j.1365-2915.2003.00420.x

[eva13194-bib-0007] Alvar, J. , Vélez, I. D. , Bern, C. , Herrero, M. , Desjeux, P. , Cano, J. , Jannin, J. , Boer, M. D. , & WHO Leishmaniasis Control Team (2012). Leishmaniasis worldwide and global estimates of its incidence. PLoS One, 7, e35671. 22693548 10.1371/journal.pone.0035671 PMC3365071

[eva13194-bib-0008] Barrett, R. D. , & Schluter, D. (2008). Adaptation from standing genetic variation. Trends in Ecology and Evolution, 23, 38–44. 18006185 10.1016/j.tree.2007.09.008

[eva13194-bib-0010] Belen, A. , Kucukyildirim, S. , & Alten, B. (2011). Genetic structures of sand fly (Diptera: Psychodidae) populations in a leishmaniasis endemic region of Turkey. Journal of Vector Ecology, 36(Suppl 1), S32–S48. 21366779 10.1111/j.1948-7134.2011.00110.x

[eva13194-bib-0011] Bell, G. , & Collins, S. (2008). Adaptation, extinction and global change. Evolutionary Applications, 1(1), 3–16. 10.1111/j.1752-4571.2007.00011.x 25567487 PMC3352406

[eva13194-bib-0136] Bell, G. , & Gonzalex, A. (2009). Evolutionary rescue can prevent extinction following environmental change. Ecol Letter, 12(9), 942–948. 10.1111/j.1461-0248.2009.01350.x 19659574

[eva13194-bib-0012] Belo, V. S. , Werneck, G. L. , Barbosa, D. S. , Simões, T. C. , Nascimento, B. W. L. , da Silva, E. S. , & Struchiner, C. J. (2013). Factors associated with visceral leishmaniasis in the Americas: A systematic review and meta‐analysis. PLoS Neglected Tropical Diseases, 7, e2182. 23638203 10.1371/journal.pntd.0002182 PMC3636096

[eva13194-bib-0013] Bolnik, D. I. , & Nosil, P. (2007). Natural selection in populations subject to a migration load. Evolution, 61, 2229–2243. 17767592 10.1111/j.1558-5646.2007.00179.x

[eva13194-bib-0014] Buerkle, A. C. , & Gompert, Z. (2013). Population genomics based on low coverage sequencing: How low should we go? Molecular Ecology, 22(11), 3028–3035. 23174005 10.1111/mec.12105

[eva13194-bib-0015] Burke, M. K. , Dunham, J. P. , Shahrestani, P. , Thornton, K. R. , Rose, M. R. , & Long, A. D. (2010). Genome‐wide analysis of a long‐term evolution experiment with *Drosophila* . Nature, 467, 587–590. 20844486 10.1038/nature09352

[eva13194-bib-0017] Catchen, J. M. , Hohenlohe, P. A. , Bernatchez, L. , Funk, W. C. , Andrews, K. R. , & Allendorf, F. W. (2017). Unbroken: RADseq remains a powerful tool for understanding the genetics of adaptation in natural populations. Molecular Ecology Resources, 17(3), 362–365. 28319339 10.1111/1755-0998.12669

[eva13194-bib-0018] Chambers, J. , & Oppenheimer, S. F. (2004). Organophosphates, serine esterase inhibition, and modeling of organophosphate toxicity. Toxicological Sciences, 77, 185–187. 14992203 10.1093/toxsci/kfh060

[eva13194-bib-0019] Chevin, L.‐M. , Lande, R. , & Mace, G. M. (2010). Adaptation, plasticity, and extinction in a changing environment: Towards a predictive theory. PLoS Biology, 8, e1000357. 20463950 10.1371/journal.pbio.1000357 PMC2864732

[eva13194-bib-0020] Chevin, L. M. , Martin, G. , & Lenormand, T. (2010). Fisher’s model and the genomics of adaptation: Restricted pleiotropy, heterogenous mutation, and parallel evolution. Evolution, 64, 3213–3231. 20662921 10.1111/j.1558-5646.2010.01058.x

[eva13194-bib-0021] Coleman, R. E. , Burkett, D. A. , Sherwood, V. , Caci, J. , Dennett, J. A. , Jennings, B. T. , Cushing, R. , Ploch, J. , Hopkins, G. , & Putnam, J. L. (2011). Impact of phlebotomine sand flies on the United States military operations at Tallil Air Base, Iraq: 6. Evaluation of insecticides for the control of sand flies. Journal of Medical Entomology, 48, 584–599. 21661320 10.1603/me10226

[eva13194-bib-0022] Comeault, A. A. , Flaxman, S. M. , Riesch, R. , Curran, E. , Soria‐Carrasco, V. , Gompert, Z. , Farkas, T. E. , Muschick, M. , Parchman, T. L. , Schwander, T. , Slate, J. , & Nosil, P. (2015). Selection on a genetic polymorphism counteracts ecological speciation in a stick insect. Current Biology, 25, 1975–1981. 26119745 10.1016/j.cub.2015.05.058

[eva13194-bib-0023] Comeault, A. A. , Soria‐Carrasco, V. , Gompert, Z. , Farkas, T. E. , Buerkle, C. A. , Parchman, T. L. , & Nosil, P. (2014). Genome‐wide association mapping of phenotypic traits subject to a range of intensities of natural selection in *Timema cristinae* . The American Naturalist, 183, 711–727. 10.1086/675497 24739202

[eva13194-bib-0024] Cowles, M. K. , & Carlin, B. P. (1996). Markov chain monte carlo convergence diagnostics: A comparative review. Journal of the American Statistical Association, 91, 883–904.

[eva13194-bib-0025] Daborn, P. J. , Yen, J. L. , Bogwitz, M. R. , Le Goff, G. , Feil, E. , Jeffers, S. , Tijet, N. , Perry, T. , Heckel, D. , Batterham, P. , Feyereisen, R. , & Wilson, T. G. (2002). A single p450 allele associated with insecticide resistance in *Drosophila* . Science, 297, 2253–2256. 12351787 10.1126/science.1074170

[eva13194-bib-0026] Danecek, P. , Auton, A. , Abecasis, G. , Albers, C. A. , Banks, E. , DePristo, M. A. , Handsaker, R. E. , Lunter, G. , Marth, G. T. , Sherry, S. T. , McVean, G. , & Durbin, R. (2011). The variant call format and VCFtools. Bioinformatics, 27(15), 2156–2158. 21653522 10.1093/bioinformatics/btr330 PMC3137218

[eva13194-bib-0027] Dapkus, D. , & Merrell, D. J. (1977). Chromosomal analysis of DDT‐resistance in a long‐term selected population of *Drosophila melanogaster* . Genetics, 87, 685–697. 414959 10.1093/genetics/87.4.685 PMC1213770

[eva13194-bib-0028] David, J.‐P. , Strode, C. , Vontas, J. , Nikou, D. , Vaughan, A. , Pignatelli, P. M. , Louis, C. , Hemingway, J. , & Ranson, H. (2005). The *Anopheles gambiae* detoxification chip: A highly specific microarray to study metabolic‐based insecticide resistance in malaria vectors. Proceedings of the National Academy of Sciences of the United States of America, 102, 4080–4084. 15753317 10.1073/pnas.0409348102 PMC554807

[eva13194-bib-0029] Denlinger, D. S. , Creswell, J. A. , Anderson, J. L. , Reese, C. K. , & Bernhardt, S. A. (2016). Diagnostic doses and times for *Phlebotomus papatasi* and *Lutzomyia longipalpis* sand flies (Diptera: Psychodidae: Phlebotominae) using the CDC bottle bioassay to assess insecticide resistance. Parasites and Vectors, 9, 212. 27083417 10.1186/s13071-016-1496-3 PMC4833940

[eva13194-bib-0030] Denlinger, D. S. , Li, A. Y. , Durham, S. L. , Lawyer, P. G. , Anderson, J. L. , & Bernhardt, S. A. (2016). Comparison of *in vivo* and *in vitro* methods for blood feeding of *Phlebotomus papatasi* (Diptera: Psychodidae) in the laboratory. Journal of Medical Entomology, 53, 1112–1116. 27297215 10.1093/jme/tjw074 PMC7289326

[eva13194-bib-0031] Denlinger, D. S. , Lozano‐Fuentes, S. , Lawyer, P. G. , Black, W. C. IV , & Bernhardt, S. A. (2015). Assessing insecticide susceptibility of laboratory *Lutzomyia longipalpis* and *Phlebotomus papatasi* sand flies (Diptera: Psychodidae: Phlebotominae). Journal of Medical Entomology, 52, 1003–1012. 26336231 10.1093/jme/tjv091 PMC4574604

[eva13194-bib-0032] Dinesh, D. S. , Das, M. L. , Picado, A. , Roy, L. , Rijal, S. , Singh, S. P. , Das, P. , Boelaert, M. , & Coosemans, M. (2010). Insecticide susceptibility of *Phlebotomus argentipes* in visceral leishmaniasis endemic districts in India and Nepal. PLoS Neglected Tropical Diseases, 4, e859. 21049013 10.1371/journal.pntd.0000859 PMC2964302

[eva13194-bib-0033] Djouaka, R. F. , Bakare, A. A. , Coulibaly, O. N. , Akogbeto, M. C. , Ranson, H. , & Hemingway, J. (2008). Expression of the cytochrome P450s, *CYP6P3* and *CYP6M2* are significantly elevated in multiple pyrethroid resistant populations of *Anopheles gambiae s.s*. from southern Benin and Nigeria. BMC Genomics, 9, 538. 19014539 10.1186/1471-2164-9-538 PMC2588609

[eva13194-bib-0034] Doha, S. , Shehata, M. G. , El Said, S. , & El Sawaf, B. (1991). Dispersal of *Phlebotomus papatasi* (Scopoli) and *P. langeroni* Nitzulescu in El Hammam, Matrouh Governorate. Egypt. Annales de Parasitologie Humaine et Comparée, 66, 69–76. 10.1051/parasite/1988632146 3421642

[eva13194-bib-0035] Edi, C. V. , Djogbénou, L. , Jenkins, A. M. , Regna, K. , Muskavitch, M. A. T. , Poupardin, R. , Jones, C. M. , Essandoh, J. , Kétoh, G. K. , Paine, M. J. I. , Koudou, B. G. , Donnelly, M. J. , Ranson, H. , & Weetman, D. (2014). *CYP6* P450 enzymes and *ACE‐1* duplication produce extreme and multiple insecticide resistance in the malaria mosquito *Anopheles gambiae* . PLoS Genetics, 10, e1004236. 24651294 10.1371/journal.pgen.1004236 PMC3961184

[eva13194-bib-0036] Elshire, R. J. , Glaubitz, J. C. , Sun, Q. , Poland, J. A. , Kawamoto, K. , Buckler, E. S. , & Mitchell, S. E. (2011). A robust, simple genotyping‐by‐sequencing (GBS) approach for high diversity species. PLoS One, 6, e19379. 21573248 10.1371/journal.pone.0019379 PMC3087801

[eva13194-bib-0037] Faraj, C. , Ouahabi, S. , Adlaoui, E. B. , Elkohli, M. E. , Lakraa, L. , Rhazi, M. E. , & Ameur, B. (2012). Insecticide susceptibility status of *Phlebotomus* (*Paraphlebotomus*) *sergenti* and *Phlebotomus* (*Phlebotomus*) *papatasi* in endemic foci of cutaneous leishmaniasis in Morocco. Parasites and Vectors, 5, 51. 22429776 10.1186/1756-3305-5-51 PMC3359231

[eva13194-bib-0038] Fawaz, E. Y. , Zayed, A. B. , Fahmy, N. T. , Villinski, J. T. , Hoel, D. F. , & Diclaro, J. W. II (2016). Pyrethroid insecticide resistance mechanisms in the adult *Phlebotomus papatasi* (Diptera: Psychodidae). Journal of Medical Entomology, 53, 620–628. 26810731 10.1093/jme/tjv256

[eva13194-bib-0039] Ferriere, R. , & Legendre, S. (2012). Eco‐evolutionary feedbacks, adaptive dynamics and evolutionary rescue theory. Philosophical Transactions of the Royal Society B‐ Biological Sciences, 368, 20120081. 10.1098/rstb.2012.0081 PMC3538448 23209163

[eva13194-bib-0040] Feyereisen, R. (1995). Molecular biology of insecticide resistance. Toxicology Letters, 82(83), 83–90. 8597150 10.1016/0378-4274(95)03470-6

[eva13194-bib-0041] ffrench‐Constant, R. H. (2007). Which came first: Insecticides or resistance? Trends in Genetics, 23, 1–4. 17125882 10.1016/j.tig.2006.11.006

[eva13194-bib-0042] ffrench‐Constant, R. H. (2013). The molecular genetics of insecticide resistance. Genetics, 194, 807–815. 23908373 10.1534/genetics.112.141895 PMC3730913

[eva13194-bib-0043] ffrench‐Constant, R. H. , Daborn, P. J. , & Goff, G. L. (2004). The genetics and genomics of insecticide resistance. Trends in Genetics, 20, 163–170. 15036810 10.1016/j.tig.2004.01.003

[eva13194-bib-0044] Gao, J. , & Scott, J. G. (2006). Role of the transcriptional repressor *mdGfi‐1* in *CYP6D1v1*‐mediated insecticide resistance in the house fly, *Musca domestica* . Insect Biochemistry and Molecular Biology, 36, 387–395. 16651185 10.1016/j.ibmb.2006.02.001

[eva13194-bib-0045] Giraldo‐Calderón, G. I. , Emrich, S. J. , MacCallum, R. M. , Maslen, G. , Dialynas, E. , Topalis, P. , Ho, N. , Gesing, S. , VectorBase Consortium , Madey, G. , Collins, F. H. , & Lawson, D. (2015). VectorBase: An updated bioinformatics resource for invertebrate vectors and other organisms related with human disease. Nucleic Acids Research, 43(Database issue), D707–D713. 25510499 10.1093/nar/gku1117 PMC4383932

[eva13194-bib-0046] Gompert, Z. , Brady, M. , Chalyavi, F. , Saley, T. C. , Philbin, C. S. , Tucker, M. J. , Forister, M. L. , & Lucas, L. K. (2019). Genomic evidence of genetic variation with pleiotropic effects on caterpillar fitness and plant traits in a model legume. Molecular Ecology, 28(12), 2967–2985. 31038777 10.1111/mec.15113

[eva13194-bib-0047] Gompert, Z. , Lucas, L. K. , Buerkle, C. A. , Forister, M. L. , Fordyce, J. A. , & Nice, C. C. (2014). Admixture and the organization of genetic diversity in a butterfly species complex revealed through common and rare genetic variants. Molecular Ecology, 23, 4555–4573. 24866941 10.1111/mec.12811

[eva13194-bib-0048] Gompert, Z. , Lucas, L. K. , Nice, C. C. , Fordyce, J. A. , Forister, M. L. , & Buerkle, C. A. (2012). Genomic regions with a history of divergent selection affect fitness of hybrids between two butterfly species. Evolution, 66(7), 2167–2181. 22759293 10.1111/j.1558-5646.2012.01587.x

[eva13194-bib-0134] Gomulkiewicz, R. , & Holt, R. D. (1995). When does evolution by natural selection prevent extinction?. Evolution, 49(1), 201–207. 10.1111/j.1558-5646.1995.tb05971.x 28593677

[eva13194-bib-0051] Gonzalez, A. , Ronce, O. , Ferriere, R. , & Hochberg, M. E. (2013). Evolutionary rescue: An emerging focus at the intersection between ecology and evolution. Philosophical Transactions of the Royal Society B, 368, 20120404. 10.1098/rstb.2012.0404 PMC3538460 23209175

[eva13194-bib-0052] Groeters, F. R. , & Tabashnik, B. E. (2000). Roles of selection intensity, major genes, and minor genes in evolution of insecticide resistance. Journal of Economic Entomology, 93, 1580–1587. 11142284 10.1603/0022-0493-93.6.1580

[eva13194-bib-0053] Guan, Y. , & Stephens, M. (2011). Bayesian variable selection regression for genome‐wide association studies, and other large‐scale problems. The Annals of Applied Statistics, 5, 1780–1815.

[eva13194-bib-0054] Hamarsheh, O. , Presbar, W. , Abdeen, Z. , Sawalha, S. , Al‐Lahem, A. , & Schönian, G. (2007). Genetic structure of Mediterranean populations of the sandfly *Phlebotomus papatasi* by mitochondrial cytochrome b haplotype analysis. Medical and Veterinary Entomology, 21, 270–277. 17897368 10.1111/j.1365-2915.2007.00695.x

[eva13194-bib-0055] Hardstone, M. C. , Leichter, C. A. , & Scott, J. G. (2009). Multiplicative interaction between the two major mechanisms of permethrin resistance, *kdr* and cytochrome P450‐monooxygenase detoxification, in mosquitoes. Journal of Evolutionary Biology, 22, 416–423. 19196389 10.1111/j.1420-9101.2008.01661.x

[eva13194-bib-0056] Hassan, F. , Dinesh, D. S. , Purkait, B. , Kumari, S. , Kumar, V. , & Das, P. (2015). Bio‐chemical characterization of detoxifying enzymes in DDT resistant field isolates of *Phlebotomus argentipes* in Bihar, India. International Journal of Medicine and Pharmaceutical Sciences (IJMPS), 5, 23–32.

[eva13194-bib-0057] Hassan, M. M. , Widaa, S. O. , Osman, O. M. , Numiary, M. S. M. , Ibrahim, M. A. , & Abushama, H. M. (2012). Insecticide resistance in the sand fly, *Phlebotomus papatasi* from Khartoum State, Sudan. Parasites and Vectors, 5, 46. 22397726 10.1186/1756-3305-5-46 PMC3314797

[eva13194-bib-0058] Hemingway, J. , Field, L. , & Vontas, J. (2002). An overview of insecticide resistance. Science, 298, 96–97. 12364782 10.1126/science.1078052

[eva13194-bib-0059] Hemingway, J. , Hawkes, N. J. , McCarroll, L. , & Ranson, H. (2004). The molecular basis of insecticide resistance in mosquitoes. Insect Biochemistry and Molecular Biology, 34, 653–665. 15242706 10.1016/j.ibmb.2004.03.018

[eva13194-bib-0060] Hemingway, J. , & Ranson, H. (2000). Insecticide resistance in insect vectors of human disease. Annual Review of Entomology, 45, 371–391. 10.1146/annurev.ento.45.1.371 10761582

[eva13194-bib-0061] Hemingway, J. , Ranson, H. , Magill, A. , Kolaczinski, J. , Fornadel, C. , Gimnig, J. , Coetzee, M. , Simard, F. , Roch, D. K. , Hinzoumbe, C. K. , Pickett, J. , Schellenberg, D. , Gething, P. , Hoppé, M. , & Hamon, N. (2016). Averting a malaria disaster: Will insecticide resistance derail malaria control? Lancet, 387, 1785–1788. 26880124 10.1016/S0140-6736(15)00417-1 PMC6215693

[eva13194-bib-0062] Hotelier, T. , Nègre, V. , Marchot, P. , & Chatonnet, A. (2010). Insecticide resistance through mutations in cholinesterases or carboxylesterases: Data mining in the ESTHER database. Journal of Pesticide Science, 35, 315–320.

[eva13194-bib-0063] Hotez, P. J. (2008). Stigma: The stealth weapon of the NTD. PLoS Neglected Tropical Diseases, 2, e230. 18446206 10.1371/journal.pntd.0000230 PMC2322832

[eva13194-bib-0065] Karakus, M. , Gocmen, B. , & Özbel, Y. (2017). Insecticide susceptibility status of wild‐caught sand fly populations collected from two leishmaniasis endemic areas in western Turkey. Journal of Arthropod‐Borne Diseases, 11, 86–94. 29026855 PMC5629309

[eva13194-bib-0066] Kasai, S. , Komagata, O. , Itokawa, K. , Shono, T. , Ng, L. C. , Kobayashi, M. , & Tomita, T. (2014). Mechanisms of pyrethroid resistance in the dengue mosquito vector, *Aedes aegypti*: Target site insensitivity, penetration, and metabolism. PLoS Neglected Tropical Diseases, 8, e2948. 24945250 10.1371/journal.pntd.0002948 PMC4063723

[eva13194-bib-0067] Kasai, S. , & Scott, J. G. (2001). A house fly gene homologous to the zinc finger proto‐oncogene *Gfi‐1* . Biochemical and Biophysical Research Communications, 283, 644–647. 11341773 10.1006/bbrc.2001.4826

[eva13194-bib-0068] Kelly‐Hope, L. , Ranson, H. , & Hemingway, J. (2008). Lessons from the past: Managing insecticide resistance in malaria control and eradication programmes. Lancet Infectious Diseases, 8, 387–389. 18374633 10.1016/S1473-3099(08)70045-8

[eva13194-bib-0069] Khalid, N. M. , Aboud, M. A. , Alrabba, F. M. , Elnaiem, D. E. , & Tripet, F. (2012). Evidence for genetic differentiation at the microgeographic scale in *Phlebotomus papatasi* populations from Sudan. Parasites and Vectors, 5, 249. 23146340 10.1186/1756-3305-5-249 PMC3503571

[eva13194-bib-0070] Khan, S. A. , Aqueel, A. , Saleem, R. Q. , Zahoor, N. , Arooj, K. , Raza, M. , Abbas, S. , Afzal, X. , Haider, M. , Ahmad, M. , Idris, M. , & Bilal, R. M. & Shahid, A. (2015). Insecticide resistance in sand flies (*Phlebotomus papatasi*) against bifenthrin and cypermethrin in Chakwal, Pakistan. European Academic Research, 3, 5349–5363.

[eva13194-bib-0071] Lande, R. (1983). The response to selection on major and minor mutations affecting a metrical trait. Heredity, 50, 47–64.

[eva13194-bib-0073] Lanfear, R. , Kokko, H. , & Eyre‐Walker, A. (2014). Population size and the rate of evolution. Trends in Ecology and Evolution, 29, 33–41. 24148292 10.1016/j.tree.2013.09.009

[eva13194-bib-0074] Lanzaro, G. C. , Alexander, B. , Mutebi, J. P. , Montoya‐Lerma, J. , & Warburg, A. (1998). Genetic variation among natural and laboratory colony populations of *Lutzomyia longipalpis* (Lutz & Neiva, 1912) (Diptera: Psychodidae) from Colombia. Memórias do Instituto Oswaldo Cruz, 93, 65–69. 9698845 10.1590/s0074-02761998000100013

[eva13194-bib-0075] Lepicard, S. , Franco, B. , de Bock, F. , & Parmentier, M. L. (2014). A presynaptic role of microtubule‐associated protein 1/Futsch in *Drosophila*: Regulation of active zone number and neurotransmitter release. The Journal of Neuroscience, 34, 6759–6771. 24828631 10.1523/JNEUROSCI.4282-13.2014 PMC6608111

[eva13194-bib-0076] Li, H. (2011). A statistical framework for SNP calling, mutation discovery, association mapping, and population genetical parameter estimation from sequencing data. Bioinformatics, 27, 2987–2993. 21903627 10.1093/bioinformatics/btr509 PMC3198575

[eva13194-bib-0077] Li, H. , & Durbin, R. (2009). Fast and accurate short read alignment with Burrows‐Wheeler transform. Bioinformatics, 26, 589–595. 10.1093/bioinformatics/btp324 PMC2705234 19451168

[eva13194-bib-0078] Li, H. , Handsaker, B. , Wysoker, A. , Fennell, T. , Ruan, J. , Homer, N. , Marth, G. , Abecasis, G. , & Durbin, R. (2009). The sequence alignment/map format and SAMtools. Bioinformatics, 25(16), 2078–2079. 19505943 10.1093/bioinformatics/btp352 PMC2723002

[eva13194-bib-0079] Li, X. , Zhu, B. , Gao, X. , & Liang, P. (2016). Overexpression of UDP‐glycosyltransferase gene UGT2B17 is involved in chlorantraniliprole resistance in *Plutella xylostella* (L.). Pest Management Science, 73(7), 1402–1409. 10.1002/ps.4469 27786405

[eva13194-bib-0080] Lowry, D. B. , Hoban, S. , Kelley, J. L. , Lotterhos, K. E. , Reed, L. K. , Antolin, M. F. , & Storfer, A. (2017). Breaking RAD: An evaluation of the utility of restriction site‐associated DNA sequencing for genome scans of adaptation. Molecular Ecology Resources, 17(2), 142–152. 27860289 10.1111/1755-0998.12635 PMC5446919

[eva13194-bib-0081] Lucas, E. R. , Miles, A. , Harding, N. J. , Clarkson, C. S. , Lawniczak, M. K. N. , Kwiatkowski, D. P. , Weetman, D. , Donnelly, M. J. , & Anopheles gambiae 1000 Genomes Consortium (2019). Whole‐genome sequencing reveals high complexity of copy number variation at insecticide resistance loci in malaria mosquitoes. Genome Research, 29(8), 1250–1261. 31345938 10.1101/gr.245795.118 PMC6673711

[eva13194-bib-0082] Lucas, L. K. , Nice, C. C. , & Gompert, Z. (2018). Genetic constraints on wing pattern variation in Lycaeides butterflies: A case study on mapping complex, multifaceted traits in structured populations. Molecular Ecology Resources, 18(4), 892–907. 29532608 10.1111/1755-0998.12777

[eva13194-bib-0083] Mallet, J. (1989). The evolution of insecticide resistance: Have the insects won? Trends in Ecology and Evolution, 4, 336–340. 21227375 10.1016/0169-5347(89)90088-8

[eva13194-bib-0084] Maroli, M. , Feliciangeli, M. D. , Bichaud, L. , Charrel, R. N. , & Gradoni, L. (2013). Phlebotomine sandflies and the spreading of leishmaniases and other diseases of public health concern. Medical and Veterinary Entomology, 27, 123–147. 22924419 10.1111/j.1365-2915.2012.01034.x

[eva13194-bib-0085] McKenzie, J. A. , & Batterham, P. (1994). The genetic, molecular and phenotypic consequences of selection for insecticide resistance. Trends in Ecology and Evolution, 9, 166–169. 21236810 10.1016/0169-5347(94)90079-5

[eva13194-bib-0086] McKenzie, J. A. , & Batterham, P. (1998). Predicting insecticide resistance: Mutagenesis, selection and response. Philosophical Transactions of the Royal Society London B Biological Sciences, 353, 1729–1734. 10.1098/rstb.1998.0325 PMC1692398 10021773

[eva13194-bib-0087] McKenzie, J. A. , Parker, A. G. , & Yen, J. L. (1992). Polygenic and single gene responses to selection for resistance to diazinon in *Lucilia cuprina* . Genetics, 130, 613–620. 1551581 10.1093/genetics/130.3.613 PMC1204877

[eva13194-bib-0088] McKinney, G. J. , Larson, W. A. , Seeb, L. W. , & Seeb, J. E. (2017). RAD seq provides unprecedented insights into molecular ecology and evolutionary genetics: Comment on Breaking RAD by Lowry et al (2016). Molecular Ecology Resources, 17(3), 356–361. 28028934 10.1111/1755-0998.12649

[eva13194-bib-0135] McLaren, W. , Gil, L. , Hunt, S. E. , Riat, H. S. , Ritchie, G. R. , Thormann, A. , & Cunningham, F. (2016). The ensembl variant effect predictor. Genome biology, 17(1), 122. 27268795 10.1186/s13059-016-0974-4 PMC4893825

[eva13194-bib-0138] McLaren, W. , Pritchard, B. , Rios, D. , Chen, Y. , Flicek, P. , & Cunningham, F. (2010). Deriving the consequences of genomic variants with the Ensembl API and SNP Effect Predictor. Bioinformatics, 26(16), 2069–2070. 20562413 10.1093/bioinformatics/btq330 PMC2916720

[eva13194-bib-0089] Meng, J. , Zhang, C. , Chen, X. , Cao, Y. , & Shang, S. (2014). Differential protein expression in the susceptible and resistant *Myzus persicae* (Sulzer) to imidacloprid. Pesticide Biochemistry and Physiology, 115, 1–8. 25307459 10.1016/j.pestbp.2014.09.002

[eva13194-bib-0090] Messer, P. W. , Ellner, S. P. , & Hairston, N. G. Jr (2016). Can population genetics adapt to rapid evolution? Trends in Genetics, 32, 408–418. 27185237 10.1016/j.tig.2016.04.005

[eva13194-bib-0091] Messer, P. W. , & Petrov, D. A. (2013). Population genomics of rapid adaptation by soft selective sweeps. Trends in Ecology and Evolution, 28, 659–669. 24075201 10.1016/j.tree.2013.08.003 PMC3834262

[eva13194-bib-0092] Morrison, A. C. , Ferro, C. , Morales, A. , Test, R. B. , & Wilson, M. L. (1993). Dispersal of the sand fly *Lutzomyia longipalpis* (Diptera: Psychodidae) at an endemic focus of visceral leishmaniasis in Colombia. Journal of Medical Entomology, 30, 427–435. 8459421 10.1093/jmedent/30.2.427

[eva13194-bib-0093] Mukhopadhyay, J. , Ghosh, K. , Ferro, C. , & Munstermann, L. E. (2001). Distribution of phlebotomine sand fly genotypes (*Lutzomyia shannoni*, Diptera: Psychodidae) across a highly heterogenous landscape. The Journal of Medical Entomology, 38, 260–267. 11296833 10.1603/0022-2585-38.2.260

[eva13194-bib-0094] Mukhopadhyay, J. , Ghosh, K. , Rangel, E. F. , & Munstermann, L. E. (1998). Genetic variability in biochemical characters in Brazilian field populations of the *Leishmania* vector *Lutzomyia longipalpis* (Diptera: Psychodidae). The American Journal of Tropical Medicine and Hygiene, 59, 893–901. 9886196 10.4269/ajtmh.1998.59.893

[eva13194-bib-0095] Mukhopadhyay, J. , Rangel, E. F. , Ghosh, K. , & Munstermann, L. E. (1997). Patterns of genetic variability in colonized strains of *Lutzomyia longipalpis* (Diptera: Psychodidae) and its consequences. The American Journal of Tropical Medicine and Hygiene, 57, 216–221. 9288819 10.4269/ajtmh.1997.57.216

[eva13194-bib-0096] Nauen, R. (2007). Insecticide resistance in disease vectors of public health importance. Pest Management Science, 63, 628–633. 17533649 10.1002/ps.1406

[eva13194-bib-0097] Neve, P. , Vila‐Aiub, M. , & Roux, F. (2009). Evolutionary‐thinking in agricultural weed management. New Phytologist, 184, 783–793. 10.1111/j.1469-8137.2009.03034.x 19780985

[eva13194-bib-0099] Oakeshott, J. G. , Farnsworth, C. A. , East, P. D. , Scott, C. , Han, Y. , Wu, Y. , & Russell, R. J. (2013). How many genetic options for evolving insecticide resistance in heliothine and spodopteran pests? Pest Management Science, 69, 889–896. 23526801 10.1002/ps.3542 PMC3818700

[eva13194-bib-0101] Orr, H. A. (2005). The genetic theory of adaptation: A brief history. Nature Reviews Genetics, 6, 119–127. 10.1038/nrg1523 15716908

[eva13194-bib-0103] Orr, H. A. , & Unckless, R. L. (2014). The population genetics of evolutionary rescue. PLoS Genetics, 10, e1004551. 25121960 10.1371/journal.pgen.1004551 PMC4133041

[eva13194-bib-0104] Orshan, L. , Elbaz, S. , Ben‐Ari, Y. , Akad, F. , Afik, O. , Ben‐Avi, I. , Dias, D. , Ish‐Shalom, D. , Studentsky, L. , & Zonstein, I. (2016). Distribution and dispersal of *Phlebotomus papatasi* (Diptera: Psychodidae) in a zoonotic cutaneous leishmaniasis focus, the Northern Negev, Israel. Plos Neglected Tropical Diseases, 10, e0004819. 27427959 10.1371/journal.pntd.0004819 PMC4948823

[eva13194-bib-0105] Palumbi, S. R. (2001). Humans as the world's greatest evolutionary force. Science, 293, 1786–1790. 11546863 10.1126/science.293.5536.1786

[eva13194-bib-0106] Parchman, T. L. , Gompert, Z. , Mudge, J. , Schilkey, F. D. , Benkman, C. W. , & Buerkle, C. A. (2012). Genome‐wide association genetics of an adaptive trait in lodgepole pine. Molecular Ecology, 21, 2991–3005. 22404645 10.1111/j.1365-294X.2012.05513.x

[eva13194-bib-0107] Pedra, J. H. , McIntyre, L. M. , Scharf, M. E. , & Pittendrigh, B. R. (2004). Genome‐wide transcription profile of field‐ and laboratory‐selected dichlorodiphenyltrichloroethane (DDT)‐resistant *Drosophila* . Proceedings of the National Academy of Sciences of the United States of America, 101, 7034–7039. 15118106 10.1073/pnas.0400580101 PMC406461

[eva13194-bib-0108] Perea, E. Z. , León, R. B. , Salcedo, M. P. , Brogdon, W. G. , & Devine, G. J. (2009). Adaptation and evaluation of the bottle assay for monitoring insecticide resistance in disease vector mosquitoes in the Peruvian Amazon. Malaria Journal, 8, 208. 19728871 10.1186/1475-2875-8-208 PMC2742550

[eva13194-bib-0109] Perera, M. D. B. , Hemingway, J. , & Karunaratne, S. H. P. P. (2008). Multiple insecticide resistance mechanisms involving metabolic changes and insensitive target sites selected in anopheline vectors of malaria in Sri Lanka. Malaria Journal, 7, 168. 18755020 10.1186/1475-2875-7-168 PMC2547111

[eva13194-bib-0137] Plummer, M. , Best, N. , Cowles, K. , & Vines, K. (2006). CODA: Convergence Diagnosis and Output Analysis for MCMC. R News, 6, 7–11.

[eva13194-bib-0110] R Core Team (2013). R: A language and environment for statistical computing. R Foundation for Statistical Computing.

[eva13194-bib-0111] Raymond, M. , & Marquine, M. (1994). Evolution of insecticide resistance in *Culex pipiens* populations: The Corsican paradox. Journal of Evolutionary Biology, 7, 315–337.

[eva13194-bib-0112] Rêgo, A. , Messina, F. J. , & Gompert, Z. (2019). Dynamics of genomic change during evolutionary rescue in the seed beetle *Callosobruchus maculatus* . Molecular Ecology, 28, 2136–2154. 30963641 10.1111/mec.15085

[eva13194-bib-0113] Rivero, A. , Vézilier, J. , Weill, M. , Read, A. F. , & Gandon, S. (2010). Insecticide control of vector‐borne diseases: When is insecticide resistance a problem? PLoS Path, 6, e1001000. 10.1371/journal.ppat.1001000 PMC2916878 20700451

[eva13194-bib-0114] Romay, M. C. , Millard, M. J. , Glaubitz, J. C. , Peiffer, J. A. , Swarts, K. L. , Casstevens, T. M. , Elshire, R. J. , Acharya, C. B. , Mitchell, S. E. , Flint‐Garcia, S. A. , McMullen, M. D. , Holland, J. B. , Buckler, E. S. , & Gardner, C. A. (2013). Comprehensive genotyping of the USA national maize inbred seed bank. Genome Biology, 14, R55. 23759205 10.1186/gb-2013-14-6-r55 PMC3707059

[eva13194-bib-0115] Roush, R. T. , & McKenzie, J. A. (1987). Ecological genetics of insecticide and acaricide resistance. Annual Review of Entomology, 32, 361–380. 10.1146/annurev.en.32.010187.002045 3545056

[eva13194-bib-0116] Saavedra‐Rodriguez, K. , Strode, C. , Flores Suarez, A. , Fernandez Salas, I. , Ranson, H. , Hemingway, J. , & Black, W. C. IV (2008). Quantitative trait loci mapping of genome regions controlling permethrin resistance in the mosquito *Aedes aegypti* . Genetics, 180, 1137–1152. 18723882 10.1534/genetics.108.087924 PMC2567363

[eva13194-bib-0117] Saeidi, Z. , Vatandoost, H. , Akhavan, A. A. , Yaghoobi‐Ershadi, M. R. , Rassi, Y. , Sheikh, Z. , Arandian, M. H. , Jafari, R. , & Sanei Dehkordi, A. R. (2012). Baseline susceptibility of a wild strain of *Phlebotomus papatasi* (Diptera: Psychodidae) to DDT and pyrethroids in an endemic focus of zoonotic cutaneous leishmaniasis in Iran. Pest Management Science, 68, 669–675. 22351603 10.1002/ps.2278

[eva13194-bib-0118] Scott, J. G. (1999). Cytochrome P450 and insecticide resistance. Insect Biochemistry and Molecular Biology, 29, 757–777. 10510498 10.1016/s0965-1748(99)00038-7

[eva13194-bib-0119] Sheehan, D. , Meade, G. , Foley, V. M. , & Dowd, C. A. (2001). Structure, function and evolution of glutathione transferases: Implications for classification of non‐mammalian members of an ancient enzyme superfamily. Biochemical Journal, 360(Pt 1), 1–16. 10.1042/0264-6021:3600001 PMC1222196 11695986

[eva13194-bib-0120] Sing, T. , Sander, O. , Beerenwinkel, N. , & Lengauer, T. (2005). ROCR: Visualizing classifier performance in R. Bioinformatics, 21(20), 3940–3941. 16096348 10.1093/bioinformatics/bti623

[eva13194-bib-0121] Surendran, S. , Karunaratne, S. H. P. P. , Adams, Z. , Hemingway, J. , & Hawkes, N. J. (2005). Molecular and biochemical characterization of a sand fly populations from Sri Lanka: Evidence for insecticide resistance due to altered esterases and insensitive acetylcholinesterase. Bulletin of Entomological Research, 95, 371–380. 16048685 10.1079/ber2005368

[eva13194-bib-0122] Tabashnik, B. E. , Carrière, Y. , Dennehy, T. J. , Morin, S. , Sisterson, M. S. , Roush, R. T. , Shelton, A. M. , & Zhao, J.‐Z. (2003). Insect resistance to transgenic Bt crops: Lessons from the laboratory and field. Journal of Economic Entomology, 96, 1031–1038. 14503572 10.1603/0022-0493-96.4.1031

[eva13194-bib-0123] Vontas, J. , Blass, C. , Koutsos, A. C. , David, J.‐P. , Kafatos, F. C. , Louis, C. , Hemingway, J. , Christophides, G. K. , & Ranson, H. (2005). Gene expression in insecticide resistant and susceptible *Anopheles gambiae* strains constitutively or after insecticide exposure. Insect Molecular Biology, 14, 509–521. 16164607 10.1111/j.1365-2583.2005.00582.x

[eva13194-bib-0124] Vontas, J. , David, J.‐P. , Nikou, D. , Hemingway, J. , Christophides, G. K. , Louis, C. , & Ranson, H. (2007). Transcriptional analysis of insecticide resistance in *Anopheles stephensi* using cross‐species microarray hybridization. Insect Molecular Biology, 16, 315–324. 17433071 10.1111/j.1365-2583.2007.00728.x

[eva13194-bib-0125] Whitten, M. J. , Dearn, J. M. , & McKenzie, J. A. (1980). Field studies on insecticide resistance in the Australian sheep blowfly, *Lucilia cuprina* . Australian Journal of Biological Sciences, 33, 725–735. 7396809

[eva13194-bib-0126] Willi, Y. , van Buskirk, J. , & Hoffmann, A. A. (2006). Limits to the adaptive potential of small populations. Annual Review of Ecology Evolution and Systematics, 37, 433–458.

[eva13194-bib-0128] World Health Organization (2010). Control of the leishmaniases. Report of a Meeting of the WHO Expert Committee on the Control of Leishmaniases. WHO Technical Report Series. http://apps.who.int/iris/bitstream/10665/44412/1/WHO_TRS_949_eng.pdf

[eva13194-bib-0129] World Health Organization (2013). Second WHO report on neglected tropical diseases. http://www.who.int/leishmaniasis/en/

[eva13194-bib-0131] Xu, Q. , Zhang, L. , Li, T. , Zhang, L. , He, L. , Dong, K. , & Liu, N. (2012). Evolutionary adaptation of the amino acid and codon usage of the mosquito sodium channel following insecticide selection in the field mosquitoes. PLoS One, 7, e47609. 23082181 10.1371/journal.pone.0047609 PMC3474719

[eva13194-bib-0132] Zhou, X. , Carbonetto, P. , & Stephens, M. (2013). Polygenic modeling with Bayesian sparse linear mixed models. PLoS Genetics, 9, e1003264. 23408905 10.1371/journal.pgen.1003264 PMC3567190

[eva13194-bib-0133] Zhu, F. , Gujar, H. , Gordon, J. R. , Haynes, K. F. , Potter, M. F. , & Palli, S. R. (2013). Bed bugs evolved unique adaptive strategy to resist pyrethroid insecticides. Scientific Reports, 3, 1456. 23492626 10.1038/srep01456 PMC3596983

