# Supplementary material for: Standing genetic variation in laboratory populations of insecticide‐susceptible Phlebotomus papatasi and Lutzomyia longipalpis (Diptera: Psychodidae: Phlebotominae) for the evolution of resistance
Source: Evol Appl. 2021 Feb 9;14(5):1248–62. doi: 10.1111/eva.13194 (PMC8127718; doi:10.1111/eva.13194)
Supplement: Supplementary file 1 — Supplementary Material [file EVA-14-1248-s001.docx]

**Table S1.** Association mapping of SNVs with the ten largest model-average point estimates from the *Phlebotomus papatasi* permethrin treatment.

| **Scaffold:Position** | **Model-Average Point Estimate** | **Posterior Inclusion Probability** | **Genetic Consequence** | **Gene** | **Gene Function** |
| --- | --- | --- | --- | --- | --- |
| 1601:26833 | 2.7706 | 0.4201 | Intergenic variant |  |  |
| 3565:10014 | 1.1012 | 0.4773 | Downstream variant | PPAI005735 | Unknown |
| 99828:233 | 0.3672 | 0.0909 | Intergenic variant |  |  |
| 67723:636 | 0.1801 | 0.1746 | Intergenic variant |  |  |
| 53775:723 | 0.1135 | 0.0874 | Intergenic variant |  |  |
| 94246:1353 | 0.1048 | 0.0754 | Intergenic variant |  |  |
| 59:92221 | 0.0988 | 0.0345 | Intergenic variant |  |  |
| 59:92218 | 0.095 | 0.0333 | Intergenic variant |  |  |
| 5534:4801 | 0.0588 | 0.0147 | Intergenic variant |  |  |
| 395:79954 | 0.057 | 0.0464 | Intergenic variant |  |  |

**Table S2.** Association mapping of SNVs with the ten largest model-average point estimates from the *Phlebotomus papatasi* malathion treatment.

| **Scaffold:Position** | **Model-Average Point Estimate** | **Posterior Inclusion Probability** | **Genetic Consequence** | **Gene** | **Gene Function** |
| --- | --- | --- | --- | --- | --- |
| 661:31493 | 0.023 | 0.0096 | Synonymous variant | PPAI009906 | Serine protease |
| 2202:3597 | 0.009 | 0.0119 | Intergenic variant |  |  |
| 48932:4971 | 0.0059 | 0.0051 | Downstream variant | PPAI008313 | Mitochondrial substrate/solute carrier |
| 5205:7108 | 0.0055 | 0.0049 | Upstream variant | PPAI008803 | Zinc finger |
| 29:84808 | 0.0043 | 0.0058 | Intergenic variant |  |  |
| 2600:10209 | 0.0039 | 0.0079 | 1. Upstream variant | 1. PPAI004239 | 1. Galactose-binding domain-like; BRCT domain |
|  |  |  | 2. Synonymous variant | 2. PPAI004240 | 2. Unknown |
| 781:30846 | 0.0036 | 0.0054 | Upstream variant | PPAI010446 | Glycoside hydrolase superfamily |
| 42:333968 | 0.00033 | 0.0064 | Intergenic variant |  |  |
| 42:333962 | 0.0033 | 0.0064 | Intergenic variant |  |  |
| 595:439 | 0.0032 | 0.0068 | Intergenic variant |  |  |

**Table S3.** Association mapping of SNVs with the ten largest model-average point estimates from the *Lutzomyia longipalpis* permethrin treatment.

| **Scaffold:Position** | **Model-Average Point Estimate** | **Posterior Inclusion Probability** | **Genetic Consequence** | **Gene** | **Function** |
| --- | --- | --- | --- | --- | --- |
| 9743:508 | 0.0193 | 0.0388 | Intergenic variant |  |  |
| 136:192398 | 0.0168 | 0.0276 | Downstream variant | LLOJ001674 | Unknown |
| 2068:4242 | 0.0149 | 0.0195 | Intergenic variant |  |  |
| 35:185239 | 0.0134 | 0.0192 | Intron variant | LLOJ005493 | Orange domain-like |
| 113:53407 | 0.0133 | 0.0134 | Intron variant | LLOJ000771 | Isoprenoid synthase |
| 2606:6769 | 0.0131 | 0.024 | Intergenic variant |  |  |
| 796:44731 | 0.0126 | 0.0156 | Intron variant | LLOJ009024 | Dwarfin |
| 486:22044 | 0.0122 | 0.0222 | Upstream variant | LLOJ006679 | Unknown |
| 2606:6737 | 0.0108 | 0.0203 | Intergenic variant |  |  |
| 142:135900 | 0.0103 | 0.0168 | 1. Downstream variant | 1. LLOJ001884 | 1. Leucine-rich repeat domain |
|  |  |  | 2. Synonymous variant | 2. LLOJ001885 | 2. Leucine-rich repeat domain; |
|  |  |  |  |  | Toll/interleukin-1 receptor homology (TIR) domain |

| **Scaffold:Position** | **Model-Average Point Estimate** | **Posterior Inclusion Probability** | **Genetic Consequence** | **Gene** | **Gene Function** |
| --- | --- | --- | --- | --- | --- |
| 31:237972 | 0.3862 | 0.0128 | 1. Downstream variant | 1. LLOJ005038 | 1. Protein disulfide isomerase |
|  |  |  | 2. 5' UTR | 2. LLOJ005039 | 2. PDCD5-related |
|  |  |  | 3. Downstream variant | 3. LLOJ005040 | 3. Nuclear envelope phosphatase-regulatory-like |
|  |  |  | 4. Upstream variant | 4. LLOJ005041 | 4. Microtubule-associated protein RP/EB |
| 25:59200 | 0.2536 | 0.0271 | Synonymous variant | LLOJ004221 | Transcription factor CP2 |
| 8:211637 | 0.2456 | 0.0088 | 1. Synonymous variant | 1. LLOJ009054 | 1. Zinc finger |
|  |  |  | 2. 5' UTR | 2. LLOJ009053 | 2. Zinc finger |
|  |  |  | 3. Upstream variant | 3. LLOJ009055 | 3. Transmembrane Fragile-X-F-associated protein |
|  |  |  | 4. Downstream variant | 4. LLOJ009056 | 4. Intra-flagellar transport protein |
| 1643:2211 | 0.108 | 0.0074 | Intergenic variant |  |  |
| 850:9150 | 0.0892 | 0.0057 | Downstream variant | LLOJ009325 | Alpha/beta hydrolase fold |
| 1282:13137 | 0.0862 | 0.0073 | Downstream variant | LLOJ001414 | Glycosyltransferase |
| 742:34072 | 0.0844 | 0.0477 | Intergenic variant |  |  |
| 875:56646 | 0.074 | 0.0275 | Intergenic variant |  |  |
| 2006:11071 | 0.0693 | 0.032 | Intergenic variant |  |  |
| 306:6417 | 0.0661 | 0.0054 | Intergenic variant |  |  |

**Table S4.** Association mapping of SNVs with the ten largest model-average point estimates from the *Lutzomyia longipalpis* malathion exposed treatment.

**Supplemental Figure Legends**

**Fig S1. Correlations between minor allele frequencies between the sand flies that survived and perished in the *P. papatasi* permethrin treatment (r= 0.985; A), *P. papatasi* malathion treatment (r = 0.987; B), *L. longipalpis* permethrin treatment (r = 0.981; C), and *L. longipalpis* malathion treatment (r = 0.968; D).**

**Fig S2.** **Heat maps showing the pairwise linkage disequilibrium (measured by r^2^) of the top 10 SNVs with the highest MAPE scores for the *P. papatasi* permethrin treatment (A), *P. papatasi* malathion treatment (B), *L. longipalpis* permethrin treatment (C), and *L. longipalpis* malathion treatment (D).**

**Fig S1.**


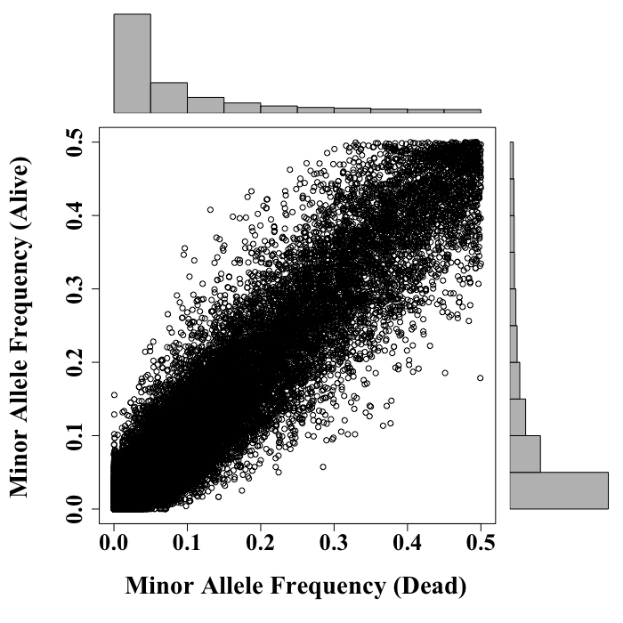

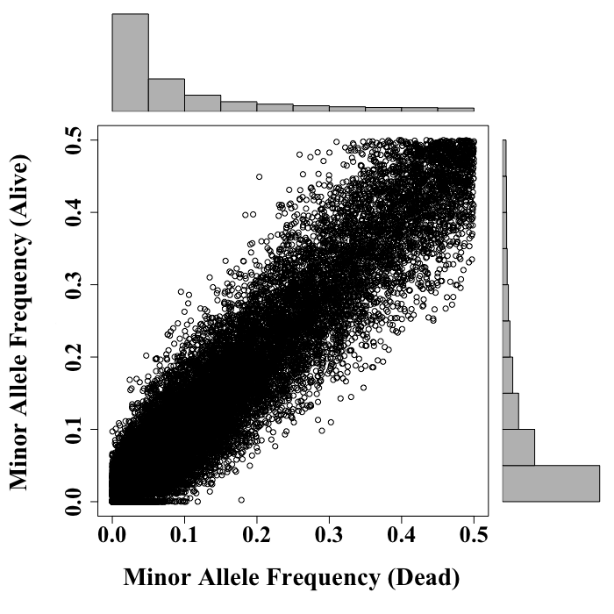
**A. B.**


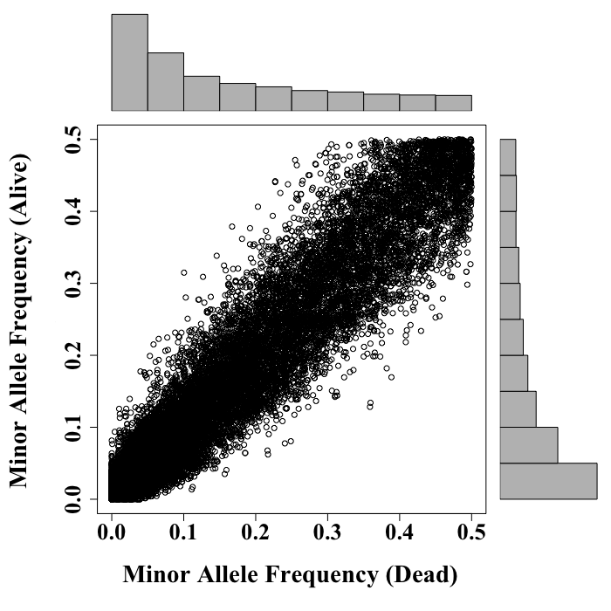

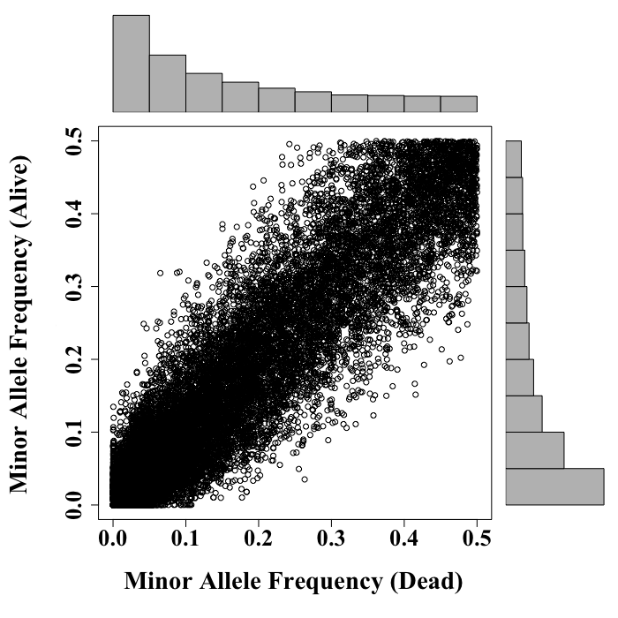
**C. D.**

**Fig S2.**


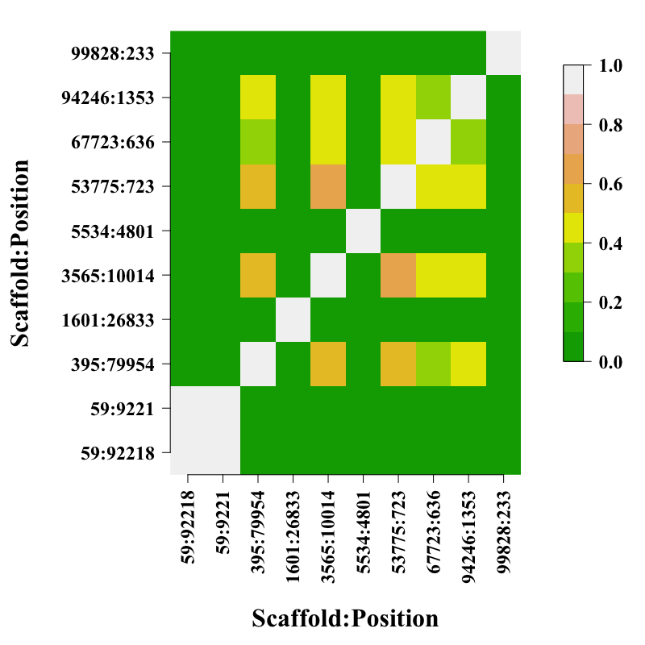

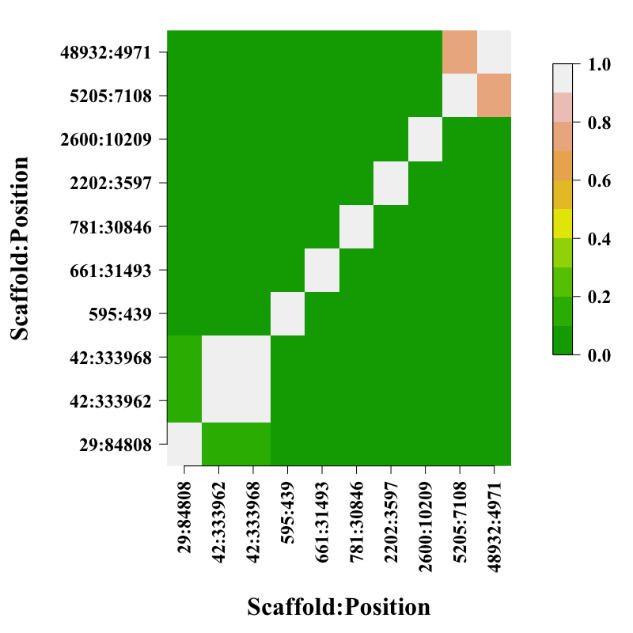
**A. B.**


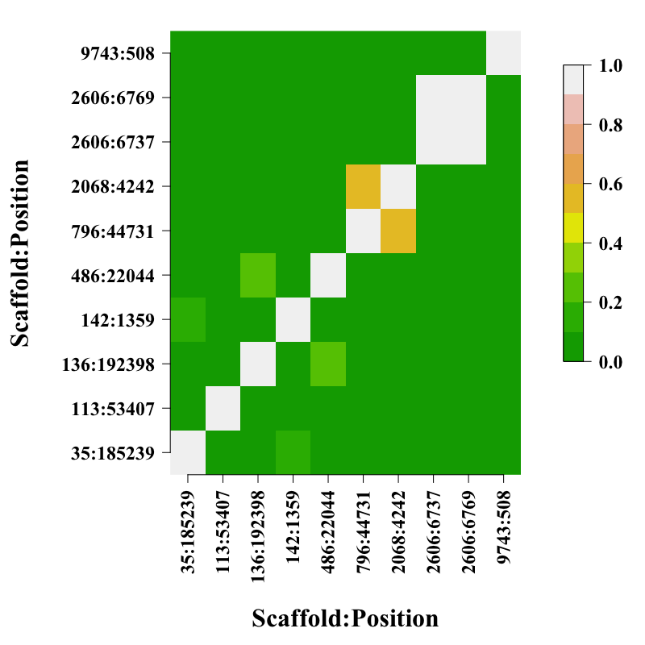

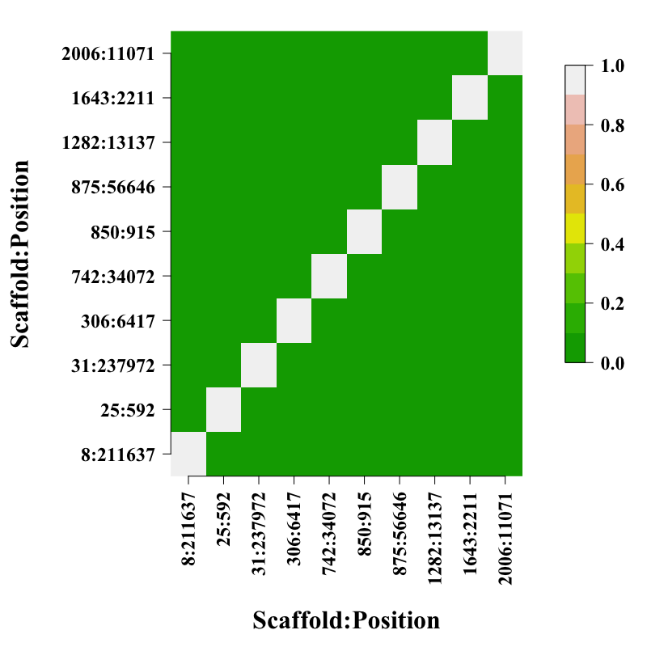
**C. D.**
